# The Mungo Mega-Lake Event, Semi-Arid Australia: Non-Linear Descent into the Last Ice Age, Implications for Human Behaviour

**DOI:** 10.1371/journal.pone.0127008

**Published:** 2015-06-17

**Authors:** Kathryn E. Fitzsimmons, Nicola Stern, Colin V. Murray-Wallace, William Truscott, Cornel Pop

**Affiliations:** 1 Department of Human Evolution, Max Planck Institute for Evolutionary Anthropology, Deutscher Platz 6, D-04103 Leipzig, Germany; 2 Archaeology Program, La Trobe University, Bundoora VIC 3086, Australia; 3 School of Earth and Environmental Sciences, University of Wollongong, Wollongong NSW 2522, Australia; Universidade do Algarve, PORTUGAL

## Abstract

The Willandra Lakes complex is one of the few locations in semi-arid Australia to preserve both paleoenvironmental and Paleolithic archeological archives at high resolution. The stratigraphy of transverse lunette dunes on the lakes’ downwind margins record a late Quaternary sequence of wetting and drying. Within the Willandra system, the Lake Mungo lunette is best known for its preservation of the world’s oldest known ritual burials, and high densities of archeological traces documenting human adaptation to changing environmental conditions over the last 45 ka. Here we identify evidence at Lake Mungo for a previously unrecognised short-lived, very high lake filling phase at 24 ka, just prior to the Last Glacial Maximum. Mega-lake Mungo was up to 5 m deeper than preceding or subsequent lake full events and represented a lake volume increase of almost 250%. Lake Mungo was linked with neighboring Lake Leaghur at two overflow points, creating an island from the northern part of the Mungo lunette. This event was most likely caused by a pulse of high catchment rainfall and runoff, combined with neotectonic activity which may have warped the lake basin. It indicates a non-linear transition to more arid ice age conditions. The mega-lake restricted mobility for people living in the area, yet archeological traces indicate that humans rapidly adapted to the new conditions. People repeatedly visited the island, transporting stone tools across water and exploiting food resources stranded there. They either swam or used watercraft to facilitate access to the island and across the lake. Since there is no evidence for watercraft use in Australia between initial colonization of the continent prior to 45 ka and the mid-Holocene, repeated visits to the island may represent a resurrection of waterfaring technologies following a hiatus of at least 20 ky.

## Introduction

Lake Mungo is the best known basin within the Willandra Lakes World Heritage Area in semi-arid Australia. Its significance is threefold. Firstly, the transverse lunette dune on its downwind margins preserves Australia’s oldest known human remains and widespread archeological traces documenting human behavioral change [1–3]. Second, the lunette’s stratigraphy provides a comprehensive record of paleoenvironmental and hydrologic change over the last full glacial cycle, in a region with poor preservation of such archives [4–6]. Finally, the conjunction of paleoenvironmental and archeological evidence presents a unique archive of human-environmental interactions over the last ca. 50 ka [6, 7]. The strategies people developed in response to changing environmental conditions, and the nature and duration of climatic transitions in the region, however, remains poorly defined [7].

**Fig 1:**
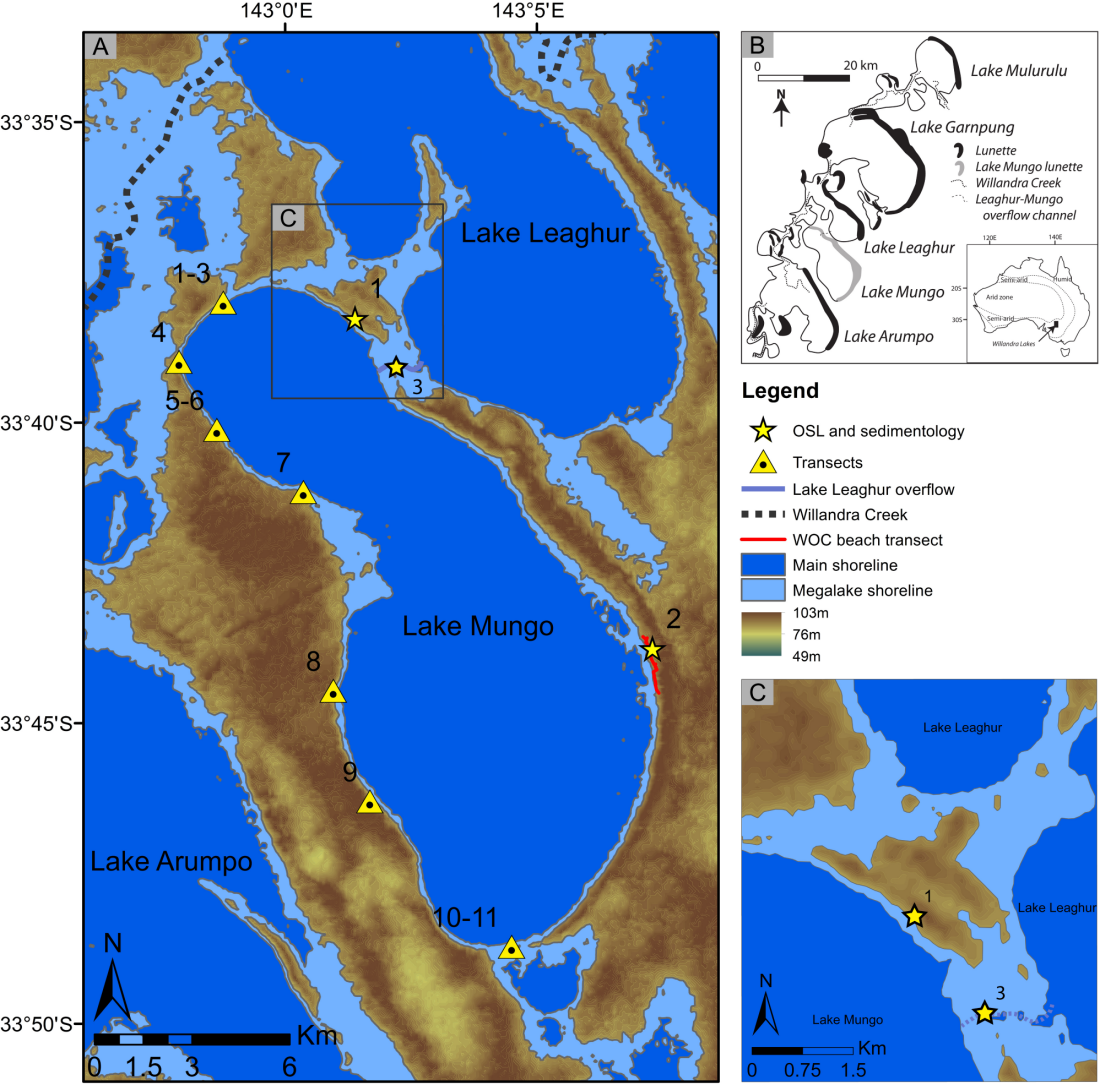
Location of Lake Mungo and the extent of its shorelines based on digital elevation models (DEMs). A. DEMs illustrating the Mungo lake shorelines during the mega-lake (75 m AHD) and main high lake phases (68–70 m AHD). The locations of surveyed transects of the shorelines are shown, including the lateral 75 m AHD beach transect (WOC beach transect), the dating and sedimentology transects (1. Northern lunette; 2. Central lunette). The location of channel sediment dating is also shown (3). B. Location of Lake Mungo within the Willandra Lakes system, and within Australia (inset). C. DEM of the northern Mungo lunette island during the mega-lake phase, showing the extent of the two connecting channels. The locations of the northern lunette transect (1) and channel dating study (3) are also shown.

Lake Mungo is an overflow lake within the presently dry Willandra Lakes system (Fig 1). Lake filling and drying responded to rainfall and runoff in the catchment headwaters—the temperate highlands of southeastern Australia, located more than 600 km to the east [8, 9]. This regime persisted until ca. 14 ka when the major inflow channel, the Willandra Creek—a paleochannel of the Lachlan River [10–12]—ceased to flow [4–6]. Since then, the Willandra lakes have remained dry. The direct connection between climate variability in the catchment headwaters, fluvial activity in the Lachlan River and Willandra Creek, and the hydrology of the Willandra Lakes, is difficult to test due to the scarcity of reliably dated paleoenvironmental evidence [11–14]. The lakes were driven by distant climatic drivers, whereas the surrounding dunefields responded to local conditions relating to the expansion and contraction of the continental arid zone [15–17].

It has been assumed that the lakes always filled to the same level, defined by the altitudes of overflow points [5]. The shoreline levels of individual lakes were determined by their relative positions within the overflow system [4, 5]. Lake Mungo was therefore assumed to fill repeatedly from its neighboring lake, Leaghur, to 68–70 m (Australian Height Datum; AHD), throughout its lifespan as an active lake [4, 5]. However, this assumption was based on limited early surveying using methods which lacked the precision now available with differential global positioning systems (dGPS). This limitation prevented accurate comparison of relative shoreline levels, and definition of the influence of neotectonic activity on lake shoreline levels through basin warping [4, 18–22].

It remains a challenge to establish what the archeological traces preserved at Lake Mungo reveal about the way in which technological, economic and social strategies changed in response to variable conditions. The poorly understood broader climatic context for landscape change is one limitation. Systematic archeological surveys aimed to quantify occupation density and subsistence strategies during different hydrologic phases began only recently [7]. The landscape palimpsest at Lake Mungo indicates that humans continuously occupied the lunette, or at least repeatedly visited the area, since arriving in the region during lake full conditions ca. 50 ka. Amongst the earliest traces of their activities are burials indicative of complex funerary practices [3]. Evidence for direct interaction between people and the lakes is limited to the presence of fish remains within hearths created during lake drying phases [4, 5, 23]. The exploitation of aquatic resources appears not to have been common during lake full periods [23], despite occupation of the lunettes at these times [6, 7].

Here we examine new evidence for a short-lived, extremely high lake phase at Lake Mungo which occurred during the transition into the last glacial maximum (LGM), during human occupation of the region. We establish that this “mega-lake” shoreline lay 5 m above the main lake full level and connected Lake Mungo with neighboring Lake Leaghur for a short period of time. We assess the possible causes of the short-lived mega-lake and its implications for climatic change. We discuss human responses to the sudden, extreme landscape changes associated with the mega-lake and the archeological traces from this period. Our results provide new insights into climatic change, human behavior and resilience in the deep past.

## Results

### Identification and characteristics of the Lake Mungo shorelines

During geological surveys of the Mungo lunette [6, 7], an additional line of beach gravels was observed which sits substantially higher than the main shoreline. We clarified the elevation of the main shoreline and compared this with the newly identified, higher elevation shoreline by surveying with dGPS; confirmed the mega-lake sediments as deriving from shoreline facies; and reconstructed the mega-lake event using a digital elevation model (DEM).

In order to quantify relative shoreline levels, we first surveyed the main Mungo shoreline using dGPS. Its elevation was previously assumed to lie at 68–70 m AHD, based on the level of the Lake Leaghur overflow channel [4, 5]. The eastern and southern shoreline comprises gently sloping gravel bands containing non-local silcrete and quartzite [4]. Erosion of the lunette has resulted in discontinuous exposure of these gravels. On the western lake margin, the main shoreline level is expressed by wave-cut benching. We surveyed the main shoreline along a 250 m transect in the southern locality of Joulni, and found that the shoreline lies higher (71 m AHD; S5 Fig) than previously postulated [5]. We identified wave-cut benching corresponding to the major shoreline at eight locations along the western lake margin. Results confirm an elevation varying between 69–72 m AHD (S1 Table; S2 Fig; S3 Fig; S4 Fig). The observed variation in shoreline elevation of 3 vertical metres across Lake Mungo provides concrete evidence for neotectonic warping of the basin over time. Variation on this scale cannot be attributed solely to wave set up under prevailing wind directional change.

**Fig 2:**
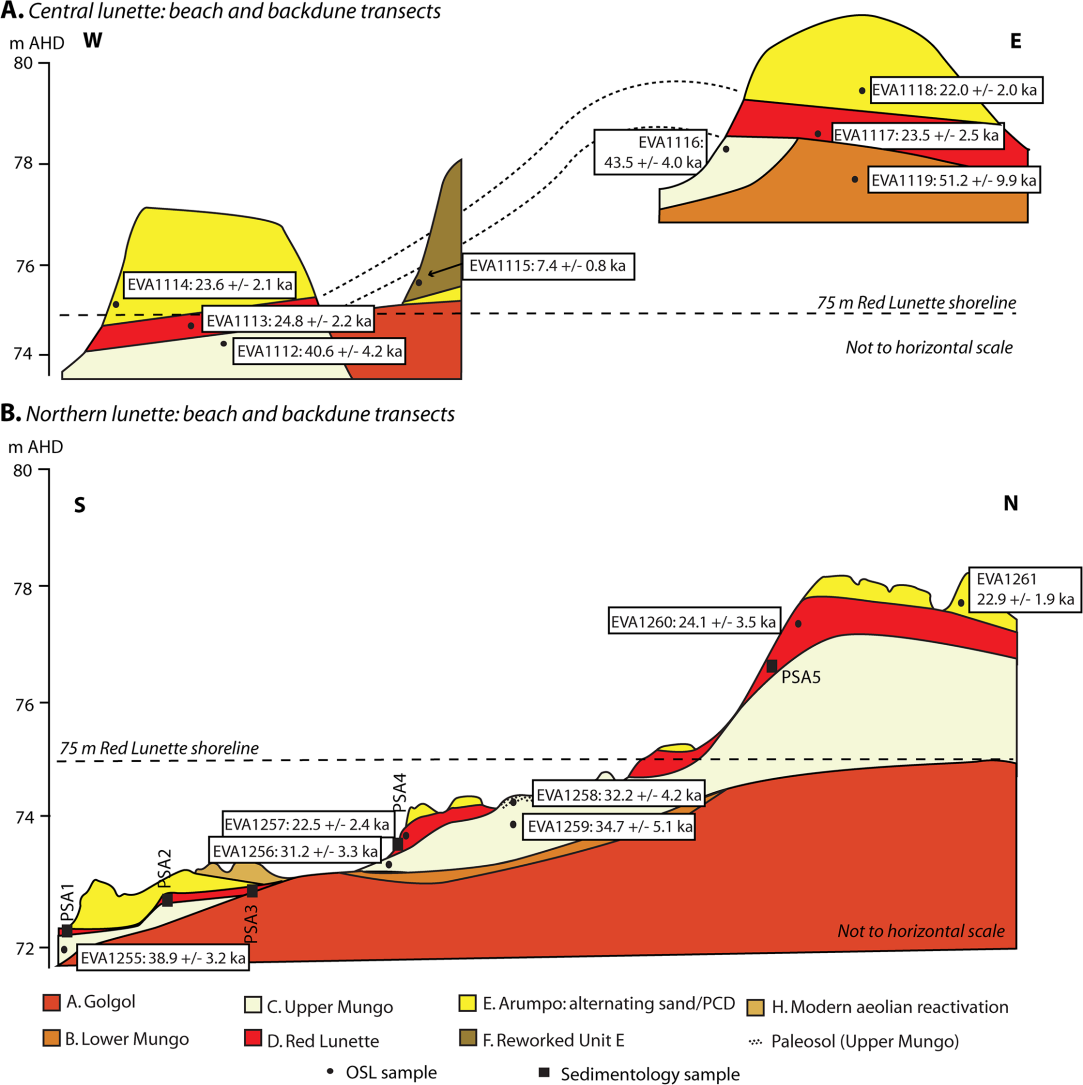
Schematic cross-sections showing the chronostratigraphy of the Lake Mungo lunette. A. Stratigraphy, OSL ages and sampling positions of the central lunette transect, linking the RL beach with its backdune. B. Stratigraphy, OSL ages and sampling positions (both for OSL dating and particle-size analyses) of the northern lunette transect. Uncertainties of the OSL ages are 1σ. The 75 m AHD shoreline is marked in both transects as a dotted line.

We then undertook surveying of the newly identified, higher shoreline level. Beach gravels associated with the mega-lake shoreline were surveyed along a 2.5 km transect of the central portion of the lunette, and along an additional transect in the northern part of the lunette. Our surveys yielded consistent elevations of 74–75 m AHD (Figs 1; 2; S1 Fig). Surveying along the western shoreline (Fig 1) identified benching consistent with wave-assisted erosion at 74–75 m AHD at four sites (S1 Table; S2 Fig; S3 Fig). These results confirm an additional, previously unidentified shoreline consistently 3–5 vertical metres above the main shoreline.

The sediments of the mega-lake shoreline lunette on the lake’s eastern and northern margins grade from beach gravels into a sandy beach foredune, which we have named the Red Lunette (RL; S8 Fig; S9 Fig). The beach gravels comprise a mixture of wave-reworked soil carbonate nodules originally of local or distal pedogenic origin, and distally-derived quartzite and silcretes (S9 Fig). The RL sandy foredune comprises subrounded red sand and forms a thin, well defined dune which can be observed in exposure at several points along the central and northern parts of the lunette. The RL foredune sands contain partially preserved iron oxide coatings (S8 Fig) which suggest local reworking of underlying iron oxide-rich units (Golgol or Lower Mungo). The RL sediments are similar in character to the Lower Mungo unit (S8 Fig; S4 Table), which also represents a lake full phase [6]. The RL sediments also contain a substantial proportion (13–28%) of silt-sized material (S10 Fig; S5 Table; “wustenquarz” [24]). The fine silts were most likely generated by coeval eolian activity in the local dunefields [16]. The RL sediment characteristics are consistent with a high lake level phase with sufficient wave energy to transport gravels around the lake.

We reconstructed the newly identified shoreline based on adjusted Shuttle Radar Topography Mission (SRTM) DEMs, combined with field dGPS data (Fig 1A). The mega-lake event resulted in overflow from Lake Leaghur at two points which reached 5 m depth in places, and included two channels >190 m wide and >2 m deep. Mega-lake flooding isolated the northernmost lunette as an island (Fig 1C).

The stratigraphy of the main channel linking Lakes Leaghur and Mungo (Fig 1A and 1C) was also investigated in order to identify subaqueously deposited sediments associated with the mega-lake phase, and to ascertain the elevation of the channel at this time. Recent gully erosion has exposed the stratigraphy at the lowest point in the landscape (70.9 m AHD). The sedimentary sequence is dominated by laminated, upward-fining packages of pale sands, interbedded with clayey sand laminae, indicative of alternating pulses of bedload transport deposited during lake filling, and fluvial-eolian infill deposited during oscillating lake levels, respectively. The sandy packages are occasionally interbedded with more clay-rich layers suggesting periodic cessation of channel activity consistent with lake drying. Within the exposed sequence, however, no single stratigraphic package could clearly be associated with the mega-lake facies. The RL-phase channel unit can therefore only be identified by correlating RL dune sediments with channel sands of the same age.

We calculated the area and volume of the main and mega-lake based on the DEM reconstructions for comparison (S2 Table). The mega-lake represents a 17% increase in lake area, and a volume increase of 249%.

### Timing and duration of the mega-lake

Stratigraphically the RL postdates, and in some places truncates, a lake drying unit (Upper Mungo). It grades upwards into alternating sands and clayey sands (Arumpo unit), which represents a phase of oscillating lake levels reaching the main shoreline level.

The timing of the mega-lake was determined by optically stimulated luminescence (OSL) dating of beach, foredune and backdune RL sediments taken from two transects in the central and northern lunette (Fig 2). Sediments both under- and overlying the RL were also dated. Single aliquot and single grain quartz ages are in agreement (Table 1) and yield a weighted mean age and standard deviation (1σ) for the RL of 23.7±1.0 ka. This indicates a relatively short-lived high lake phase.

**Table 1:** Equivalent dose (D_e_), dose rate data and OSL age estimates for the Lake Mungo lunette transects and Leaghur Channel samples.

Sample	Depth (m)	D_e_ (Gy)	Dose rate data (Gy/ka)				Age (ka)
			Gamma^d^	Beta^e^	Cosmic	Total	
***Red lunette beach–central lunette***							
EVA1112	1.8±0.1	38.9±1.1^a^	0.30±0.03	0.49±0.05	0.12±0.01	0.91±0.09	42.9±4.5
*EVA1112*	1.8±0.1	*36*.*8*±*0*.*8* ^*a*^	0.30±0.03	0.49±0.05	0.12±0.01	0.91±0.09	*40*.*6±4*.*2*
**EVA1113**	**1.2±0.1**	**23.4±0.6** ^**a**^	**0.31±0.03**	**0.49±0.05**	**0.13±0.01**	**0.92±0.08**	**25.3±2.3**
***EVA1113***	**1.2±0.1**	***22*.*9*±*0*.*4*** ^***a***^	**0.31±0.03**	**0.49±0.05**	**0.13±0.01**	**0.92±0.08**	***24*.*8*±*2*.*2***
EVA1114	1.3±0.1	24.4±0.8^a^	0.34±0.03	0.56±0.06	0.12±0.01	1.02±0.08	23.9±2.1
*EVA1114*	1.3±0.1	*24*.*1*±*0*.*8* ^*a*^	0.34±0.03	0.56±0.06	0.12±0.01	1.02±0.08	*23*.*6*±*2*.*1*
EVA1115	1.3±0.1	11.1±1.0^a^	0.24±0.02	0.36±0.04	0.12±0.01	0.72±0.08	15.4±2.1
*EVA1115*	1.3±0.1	*5*.*3*±*0*.*2* ^*b*^ ^,^ ^*c*^	0.24±0.02	0.36±0.04	0.12±0.01	0.72±0.08	*7*.*4*±*0*.*8*
*EVA1115*	1.3±0.1	*12*.*9*±*0*.*5* ^*b*^	0.24±0.02	0.36±0.04	0.12±0.01	0.72±0.08	*17*.*9*±*2*.*0*
***Red lunette backdune–central lunette***							
EVA1116	4.0±0.1	42.5±1.6^a^	0.33±0.03	0.50±0.05	0.09±0.01	0.92±0.08	46.4±4.5
*EVA1116*	4.0±0.1	*39*.*8*±*0*.*9* ^*a*^	0.33±0.03	0.50±0.05	0.09±0.01	0.92±0.08	*43*.*5*±*4*.*0*
**EVA1117**	**2.4±0.1**	**19.7±0.5** ^**a**^	**0.25±0.03**	**0.37±0.04**	**0.11±0.01**	**0.72±0.08**	**27.2±2.9**
***EVA1117***	**2.4±0.1**	***17*.*0*±*0*.*3*** ^***a***^	**0.25±0.03**	**0.37±0.04**	**0.11±0.01**	**0.72±0.08**	***23*.*5*±*2*.*5***
EVA1118	2.8±0.1	21.2±0.7^a^	0.32±0.03	0.50±0.05	0.10±0.01	0.92±0.08	23.0±2.1
*EVA1118*	2.8±0.1	*20*.*3*±*0*.*4* ^*a*^	0.32±0.03	0.50±0.05	0.10±0.01	0.92±0.08	*22*.*0*±*2*.*0*
EVA1119	4.5±0.1	17.5±0.7^a^	0.11±0.01	0.15±0.01	0.08±0.01	0.34±0.07	51.5±10.1
*EVA1119*	4.5±0.1	*17*.*4*±*0*.*7* ^*a*^	0.11±0.01	0.15±0.01	0.08±0.01	0.34±0.07	*51*.*2*±*9*.*9*
***Red lunette transect–northern lunette***							
EVA1255	1.3±0.1	*42*.*8*±*1*.*1* ^*a*^	0.40±0.04	0.58±0.06	0.12±0.01	1.10±0.09	*38*.*9*±*3*.*2*
EVA1256	1.0±0.1	*23*.*4*±*0*.*7* ^*a*^	0.26±0.03	0.36±0.04	0.13±0.01	0.75±0.08	*31*.*2*±*3*.*3*
**EVA1257**	**0.6±0.1**	***28*.*7*±*2*.*0*** ^***b***^	**0.53±0.05**	**0.61±0.06**	**0.14±0.01**	**1.27±0.10**	***22*.*5±2*.*4***
EVA1258	0.7±0.1	*18*.*7*±*0*.*5* ^*a*^	0.20±0.02	0.25±0.03	0.13±0.01	0.58±0.07	*32*.*2*±*4*.*2*
EVA1259	1.2±0.1	*17*.*3*±*0*.*5* ^*a*^	0.18±0.02	0.19±0.02	0.12±0.01	0.50±0.07	*34*.*7*±*5*.*1*
**EVA1260**	**0.5±0.1**	***21*.*6±2*.*4*** ^***a***^	**0.32±0.03**	**0.44±0.04**	**0.14±0.01**	**0.89±0.08**	***24*.*1±3*.*5***
EVA1261	1.8±0.1	*23*.*4±0*.*6* ^*a*^	0.36±0.04	0.54±0.05	0.12±0.01	1.02±0.08	*22*.*9±1*.*9*
***Leaghur Channel***							
EVA1265	1.5±0.1	*23*.*2±0*.*7* ^*a*^	0.29±0.03	0.35±0.04	0.12±0.01	0.76±0.08	*30*.*7±3*.*5*
EVA1269	1.0±0.1	*39*.*8±1*.*0* ^*a*^	0.74±0.07	1.26±0.13	0.13±0.01	2.12±0.17	*18*.*8±1*.*6*

Single aliquot results are shown in plain text; single grain results in italics. Water contents of 5 ± 3% were used for all samples, with the exception of the Leaghur Channel sands (3 ± 2%). Red lunette ages are shown in bold type.

^a^ Calculated using the central age model of Galbraith et al. (1999).

^b^ Calculated using the finite mixture model of Galbraith et al. (1999).

^c^ This younger component represents 47% of the total population using finite mixture modelling, and most likely reflects the most recent reactivation phase, and therefore the true age of this unit.

^d^ Attenuated; determined using high resolution germanium gamma spectrometry.

^e^ Attenuated; determined using beta counting.

The timing of channel sediment deposition was likewise determined by OSL dating of the lower and middle sediment units within exposed channel sediments linking Lakes Leaghur and Mungo, to ascertain channel elevation at the time of the mega-lake, and consequently its maximum depth. Single grain quartz ages indicate sandy fluvial-eolian deposition at a depth of 1.5 m below the present-day surface (69.4 m AHD) at 30.7±3.5 ka, and pulse of subaqueous sandy bedload deposition 1.0 m below the surface (69.9 m AHD) at 18.8±1.6 ka (Table 1). Several sandy laminae too thin for sampling lie between these two samples; at least one of these must correlate with the 23.7±1.0 ka age of the megalake, but cannot be distinguished.

The channel sediments associated with the mega-lake phase must therefore have been deposited at an elevation somewhere between 69.4–69.9 m AHD. Since the mega-lake shoreline reached 74–75 m AHD, this implies a maximum channel depth within the main channel of at least 4 m. This depth has corresponding implications for human mobility across the channels during the mega-lake.

**Fig 3:**
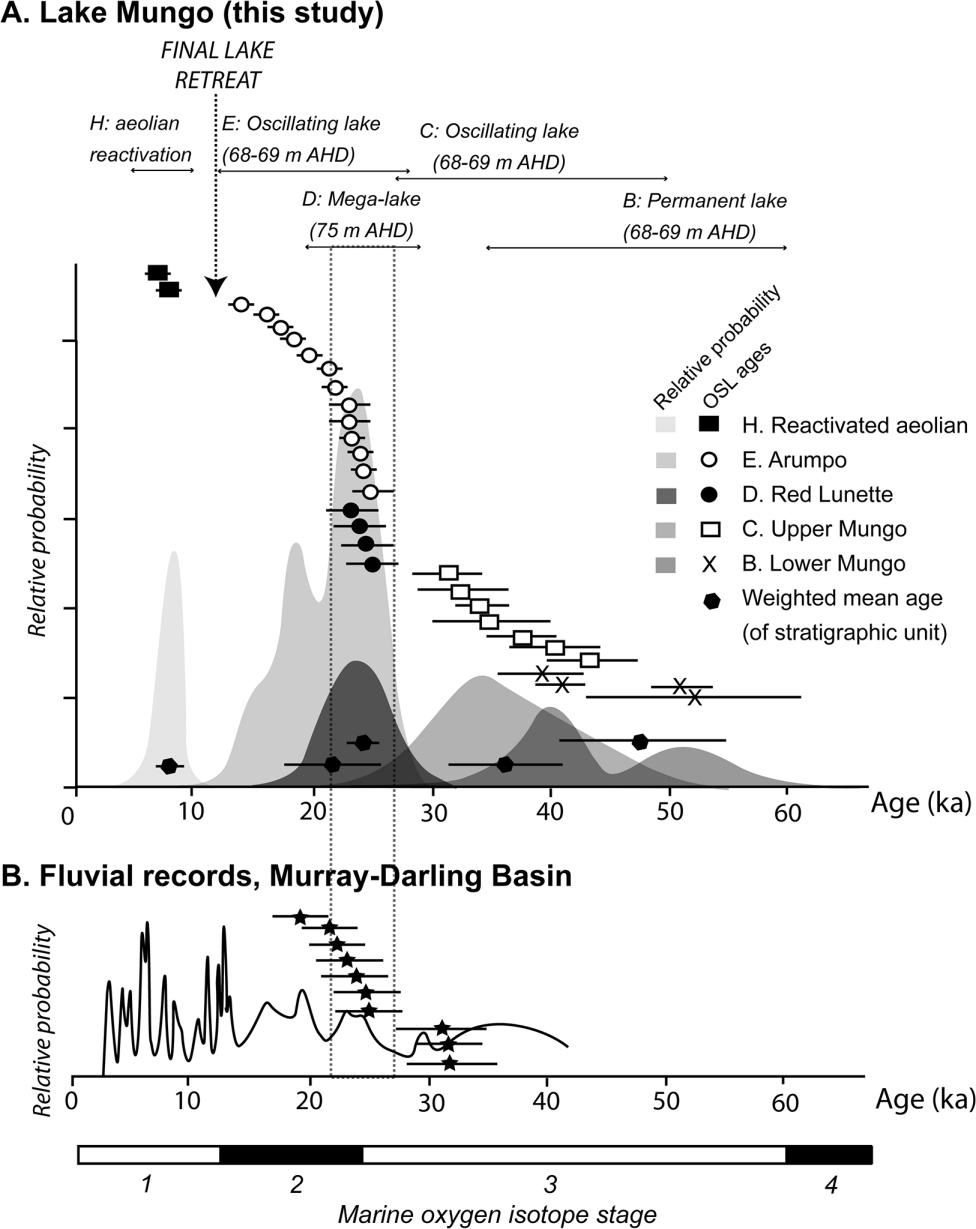
Lake Mungo chronostratigraphy and palaeohydrology, compared with palaeoenvironmental conditions in the Murray-Darling Basin (MDB). The likely duration of the mega-lake phase (within 2σ of its weighted-mean age) is highlighted by dotted lines. A. Age-ranked chronology and relative probability distributions for the stratigraphic units at Lake Mungo, based on combined OSL ages from this study and [6]. The numbers of age estimates are: Reactivated Unit H (2), Arumpo (13), RL (4), Upper (7), Lower Mungo (4). The interpreted duration of the different stratigraphic units, and the palaeoenvironmental summary, is shown at the top of the diagram. The weighted-mean ages for each of the units are shown at the base. Uncertainties of the OSL ages are 1σ. B. Probability density distribution of sandy bedload units from MDB rivers (N = 73; after [16]). Age-ranked individual OSL ages for increased fluvial activity in the Lachlan River (N = 10; [15]) are also shown. Global marine oxygen isotope chronozones [53] are shown for context.

The mega-lake immediately precedes the LGM phase of oscillating lake filling and drying associated with the Arumpo unit [6]. The weighted mean age of the Arumpo unit is 21.8±3.4 ka (1σ). Although the older part of this range overlaps with the RL, five of the 16 Arumpo samples dated yield ages more than 2σ younger than the RL (Fig 3A), indicating that the Arumpo sequence is distinctly younger, and of longer duration, than the mega-lake.

The mega-lake followed a depositional hiatus indicated by truncation of the underlying Upper Mungo unit. The unconformity suggests dry lake conditions due to lack of inflow from the Willandra Creek. Prior to the depositional hiatus, oscillating lake levels and drying conditions persisted between 43–31 ka (weighted mean age 36.6±4.6 ka).

We also provide new ages for the older lake-full Lower Mungo unit (51.2±9.9 ka), and for eolian reactivation of the lunette following final lake retreat (7.4±0.8 ka)(Fig 2).

The mega-lake phase was therefore of relatively short duration, initiated after a depositional hiatus associated with lake dry conditions, and immediately preceded the final phase of lake filling (to the main shoreline level) and drying (Fig 3A). Its timing coincides with the transition from the MIS 3 interstadial into the early glacial conditions of MIS 2.

### Archaeological occupation during the mega-lake

The mega-lake event postdates human arrival in the region, which took place during the Lower Mungo lake full phase at least 20 ka earlier [3]. A 2 km survey area in the central Mungo lunette shows that the RL contains traces of past human activity, mostly in the form of baked sediment hearths, few of which preserve associated animal remains [7].

**Fig 4:**
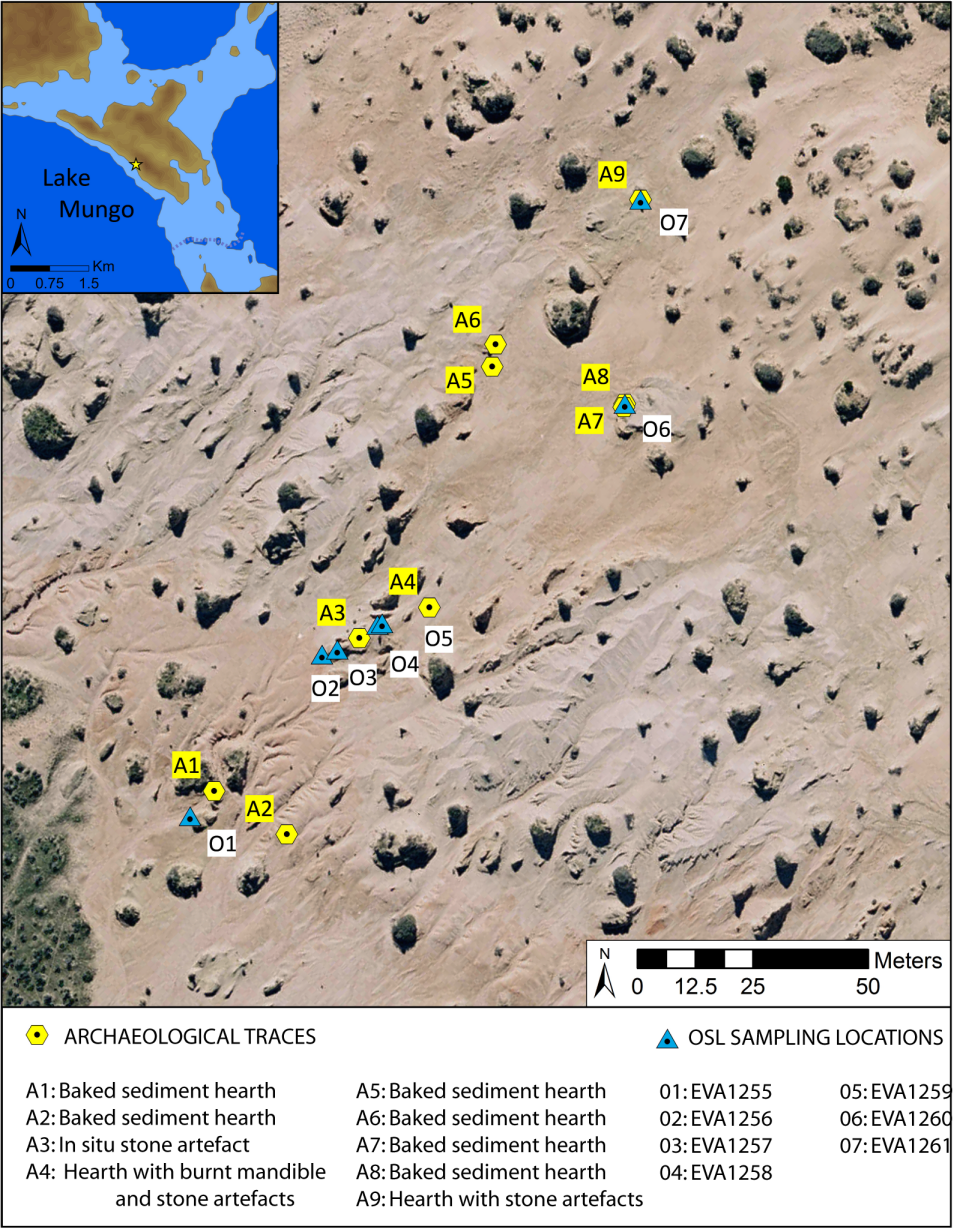
Surveyed archaeological traces and OSL sampling sites in the northern, former island, transect. A number of these traces are illustrated in the Supplementary Information. The context for A3 and A4 are shown in S17A Fig; the baked sediment hearthstones are shown in detail in S17B Fig, and its full assemblage in S17C Fig; A8 is shown in S17D Fig; the context for A5 and A6 is pictured in S17E Fig; A7 and A8 are shown in S18 Fig.

More varied archeological traces were observed within the RL on the island that formed between the two inflow channels from Lake Leaghur (Figs 1C; 4). In situ archeological traces include baked sediment hearths and carbonate hearth stones, clusters of burned and fragmented animal bones and fish otoliths, and stone artefacts (S17 Fig). In the survey transect on this former island, 8 hearths and 5 stone artefact scatters were identified in situ (Fig 4). At one locality, two hearths were observed at different elevations within the RL dune stratigraphy, with 13 cm of sediment accretion between them (S18 Fig). This indicates repeated human exploitation of food resources on the island. The greater diversity of activity traces on the island compared to the central lunette suggests that stranded prey rendered the island an attractive hunting ground. However, it is also conceded that there were additional advantages to visiting the island, not least being the substantially reduced travelling distance and time involved in passing over the island in order to travel between the western and eastern shorelines, compared with travelling by foot via the southern perimeter.

In situ stone artefacts have been observed in the RL sediments both on the island (Fig 4) and on the main lunette east of the lake. There is no raw material source for stone tool manufacture at either of these localities, implying transport of raw stone from a distant quarry or deposit. Although the raw (silcrete) material source cannot be identified, the nearest possible sources are located on the Lake Mungo western shoreline or further to the west or north.

## Discussion

### Causes of the mega-lake event

The mega-lake event indicates a substantial, short-lived, rapid rise in shoreline level up to 5 vertical metres above the main lake shoreline, around 24 ka. The two most likely drivers for this event are climate and neotectonic warping of the lake basin. Climate is more likely to have been the main influence on the mega-lake event, although there was probably interplay between the two factors.

The flooding of Lake Mungo to mega-lake level implies a higher availability of water in the catchment due to increased precipitation and runoff efficiency. The rise in shoreline level up to 5 vertical metres above the main shoreline–and corresponding increase in lake volume above main lake full levels by 249%—suggests that the flooding process was a sustained, if short-lived, event rather than the result of seasonal flooding. Fluvial and lacustrine archives within southeastern Australian catchments also indicate that more water was available in the hydrologic system up to 24 ka [11–13, 25–27]. Larger river channels and higher bedloads in the Lachlan [12], and in other rivers of the Murray-Darling system [13], peaked just prior to the mega-lake event (Fig 3B). Lake Urana, on the Riverine Plain upstream of Lake Mungo, also filled at this time [28].

The greater availability of water during the mega-lake event was accompanied by decreasing temperatures corresponding to the initial onset of the MIS 2 glacial phase. This is indicated by a transition to glacial conditions [29] and periglaciation of highland regions neighboring the Willandra/Lachlan catchment headwaters [30, 31].

The additional influence of neotectonic activity on the Mungo shorelines through basin warping cannot be precluded [18, 19, 21]. Variation in elevation of the main gravel and wave-cut benching shoreline across Lake Mungo by up to three vertical meters indicates that some degree of warping, potentially over multiple events, has influenced basin morphology during the late Pleistocene. Seismic warping has been identified in the form of morphotectonic lineations across southeastern Australia [19, 21, 32, 33], and has affected lake basin morphology at sites south and west of Lake Mungo [21, 34]. However, at present the magnitude of seismic activity and its mechanism is poorly understood [22]. Neotectonic activity is unlikely to have been the main cause of mega-lake shoreline level, since its elevation is consistent across the basin, and the event was so short-lived. Therefore, while neotectonic influence is possible, climate is more likely the main driver of mega-lake flooding.

### Implications for climate

The timing of the mega-lake at 24 ka corresponds to the early transition into the LGM. The sedimentary characteristics of the RL yield a snapshot of both distant and local climatic conditions prevailing at this time. Lake Mungo filled in response to a pulse of increased precipitation and runoff in the distant headwaters [12, 13]. At the same time, local eolian activity in the dunefields [16, 17] and distal dust transport [35, 36] intensified. The event took place against a backdrop of decreasing temperatures [13, 14]. Collectively this evidence suggests coeval humid, cooling conditions in the temperate highlands, with aridification and cooling–and expansion of the arid zone–on the semi-arid desert margins where the Willandra Lakes are located. The apparent disparity in rainfall regimes across the temperate latitudes of southeastern Australia contrasts with the prevailing view of the Australian LGM as a simple descent into cold, arid climates [8, 17, 37]. Evidence from the Mungo mega-lake suggests that the transition into the LGM was not only non-linear, but also spatially variable.

### Implications for human behavior

The sudden flooding of Lake Mungo during human occupation of the area indicates highly flexible responses to short-lived, extreme conditions. Occupation of the Mungo lunette was continuous since the first arrival of humans to the region from ca. 50 ka, through subsequent lake level oscillations including the mega-lake event, until the present dry lake conditions [3, 6]. Potential effects of the mega-lake on people are manifold, with implications for mobility in the landscape–including navigation across water–and the accessibility of food resources.

Mobility across the landscape around Lake Mungo was substantially reduced during the mega-lake event. The flooding of Mungo separated the western shoreline from the eastern lunette by >600 m of water, including a 190 m wide channel >2 m deep (Fig 1). Access across the lake bed (e.g. [38]) or around the northern lake margins would no longer have been possible. The distances needed to reach the surrounding open plains to access large mammalian prey or high quality raw materials for stone tool manufacture would have increased substantially.

Mega-lake Mungo nevertheless remained an integral part of the settlement system, which raises questions as to how people moved around the flooded landscape. Key evidence lies in the occurrence of hearths and stone artefacts within RL sediments on the island. The preservation of multiple hearths at different stratigraphic levels within the RL (Fig 4; S18 Fig) indicates repeated exploitation of food resources on the island, rather than a single opportunistic visit. The occurrence of stone artefacts in the RL sediments both on the island and on the main lunette, given that there is no raw material source at either location, indicates that the stone must have been transported to the island and lunette from a distant source, whether from the western side of the lake or further west or north. Regardless of the exact location of the source, raw stone material found on the main lunette must have been transported either across the flooded channels via the island, or for substantially greater distances around the southern margins of the lake.

The presence of both hearths and stone artefacts on the island provides concrete evidence that people repeatedly crossed the inflow channel, carrying their tools and hunting equipment with them. The two possibilities for crossing to the island are swimming and transportation by watercraft. Ethnographic data suggests that Aboriginal Australians occasionally transported stone tools in their hair or in small bags [39–42], making swimming while carrying stone tools a feasible solution. However, the more parsimonious hypothesis is that some form of watercraft was adopted as a means of transport for repeated visits to the island.

The likely use of watercraft to travel across the mega-lake–an event which followed a dry lake phase [6]–implies a highly flexible response to the sudden change in conditions, and possibly a resurrection of boat technology. While Aboriginal Australians deliberately migrated between Sunda and Sahul by boat some time prior to 45 ka [43], and regularly used watercraft to navigate coastal regions from the mid-Holocene [44], there is a distinct lack of evidence for pelagic fishing and navigation to offshore islands around the Australian coast for the approximately 40 ky following initial colonization until the mid-Holocene [45]. This has led to the assumption that watercraft technologies were abandoned after initial arrival and dispersal on the continent [45, 46]. If waterfaring was adopted to counter the challenges to human mobility about the landscape during mega-lake Mungo at 24 ka, then this represents the resurrection of a technology which had apparently been abandoned across the continent 20 ky earlier–at a location well inland and far from any major navigable rivers in the north of Australia. The inference from mega-lake Mungo at 24 ka is therefore that either boat technologies persisted in and were dispersed throughout the semi-arid zone after colonization and were co-opted to mega-lake conditions, or that people developed new technologies for water navigation in response to the sudden change in conditions. Given the distances in time and space involved, we speculate that the latter is more likely, but concede that a lack of more concrete evidence prevents confirmation at present.

## Materials and Methods

Permission to undertake fieldwork in the Willandra Lakes World Heritage Area was granted by the Technical and Scientific Advisory Committee of the Willandra Lakes World Heritage Area, the Elders' Council of the Paakantyi/Barkindji, Ngiyampaa and Mutthi Mutthi, and the Office of Environment and Heritage (New South Wales National Parks and Wildlife Service).

### Mapping, survey and field sampling

The mega-lake shoreline, and its associated sandy RL dune, was identified during stratigraphic mapping of the central part of the lunette. Mapping was undertaken using ground-truthing methods previously described [6], with air photos used as a base map and confirmed using hand-held GPS and differential GPS (dGPS) surveys georectified to Geodetic Datum of Australia (GDA) 1994, MGA zone 54, using the Australian Height Datum (AHD). Stratigraphic maps were then integrated with the locations of archeological traces recorded in georectified three-dimensional space using either total station or dGPS [6]. The elevation of the mega-lake shoreline was confirmed by dGPS surveying of beach gravel exposures along a 2.54 km transect of the central part of the lunette. Topographic surveys of the western and southern Lake Mungo shoreline were undertaken at 11 locations using dGPS to identify wave-cut benching during high lake phases. Erosion of the lunette has resulted in variable preservation of stratigraphic units along its length [6]. Thin sandy units such as the Lower Mungo and RL are particularly susceptible to erosion, which has partially removed these units from the stratigraphic record. Therefore we selected the two locations along the lunette where the RL was best exposed in cross section. These central and northern lunette transects were surveyed using dGPS and formed the focus of sedimentological and chronological investigations.

### Lake shoreline reconstruction from digital elevation models

Shorelines were reconstructed in ESRI ArcMap from SRTM DEMs (v4.1) provided by CIAT [47], reprojected to the GDA, MGA zone 54 using bilinear resampling. The greater accuracy of the dGPS data and DEMs derived from high-resolution (1m) stereo air photos (GDA 1994, MGA zone 54, AHD) was used to cross-check the accuracy of the SRTM DEM data. We found that the SRTM DEMs systematically overestimated the elevations by an average of 2.7 m (SD = 1.84) over a sampled area of approximately 25.2 km^2^ (S1 File). Therefore, we partially corrected for this systematic error by flooding the DEM to 1 m higher than the lake level elevations determined using dGPS. While conservative, this correction factor was adopted because we felt that, given the fluctuations in accuracy of the SRTM elevation data, it provided the most reliable minimum estimate for the extent of the mega-lake.

### Sedimentology

Samples for micromorphology, were collected from different stratigraphic units along the central lunette transect. Oriented samples were collected by driving 10 cm-long sections of rectangular 10x5 cm plastic piping into cleaned exposures. Thin sections were prepared by impregnation of the samples with polyester resin. Each thin section was analyzed using previously described methods [48] using an optical petrographic microscope with polarized light. Mineralogy, grain shape and roundness, pedogenic development, and the presence or absence of clay pellets were documented (S4 Table). Samples for particle-size analysis were collected from the northern transect along the RL catena from subaqueous to backdune (S5 Table). Particle-size was analyzed using a Malvern Mastersizer 2000 laser particle size analyzer at 0.25 Ф size intervals, with a minimum diameter for analyses of 0.02 μm (S10 Fig). Samples underwent minimal processing to prevent the breakdown of clay aggregates; water was gently added to the samples, which were then slowly swirled within beakers prior to analysis.

### OSL dating

OSL dating was undertaken using previously published methods [6]. The aim was to provide a comprehensive chronostratigraphy for the Red Lunette and its bracketing units along the two transects, and to ascertain the depth of the channel bed during the mega-lake relative to the present day (Table 1). Samples were collected by driving 4 cm diameter, 10 cm long stainless steel tubes horizontally into cleaned exposures, with additional material surrounding the tubes collected in sealed plastic bags for dose rate analyses. OSL samples were processed under low intensity red light; sand sized quartz (180–212 μm) was isolated using a range of chemical and physical techniques [6]. Samples from the central transect were measured using both single aliquots (1 mm mask; 24 aliquots per sample) and single grains (>600 grains per sample). Single grains only were measured for the northern transect samples. Equivalent dose (D_e_) measurements were undertaken using an automated Risø TL-DA-20 reader with a single grain laser attachment [49, 50], using the single-aliquot regenerative-dose (SAR) protocol [51, 52]. Individual grains were selected for analysis based on luminescence characteristics [6]. D_e_ was determined using either the central age [53] or finite mixture model [54]. The Supporting Information provides additional data regarding assessments of sample reliability for dating (S6 Table; S7 Table; S8 Table; S11 Fig; S12 Fig; S13 Fig; S14 Fig; S15 Fig; S16 Fig). Dose rates were determined using beta counting and high resolution germanium gamma spectrometry (the latter undertaken at the VKTA laboratory in Dresden, Germany) converted to dose rates [55], incorporating present-day moisture content with a large error to allow for temporal variability [56], added to the cosmic ray component [57].

## Supporting Information

S1 Fig Confirmation of the elevation consistency of the Red Lunette mega-lake shoreline at ca. 75 m AHD, based on dGPS measurements taken during ground truthing. Clockwise from top left: Location of the transect within the Lake Mungo lunette; transect from ground truthing, based on observation of shoreline features; lateral view of consistency of shoreline elevation, projected onto the digital elevation model generated from the aerial photos collected from the central portion of the lunette. Note that the dGPS data and aerial photo data are more accurate than the shoreline reconstruction based on the SRTM data, which accounts for the divergence between the directly surveyed shoreline and that shown in the map top right.
(TIF)

S2 Fig Transect cross-sections 1–4 for the northwestern Lake Mungo shoreline. Inset shows the location of these transects relative to the reconstructed main (ca. 70–71 m AHD) and mega-lake (ca. 75 m AHD) shorelines.
(TIF)

S3 Fig Transect cross-sections 5–7 for the western Lake Mungo shoreline. Inset shows the location of these transects relative to the reconstructed main (ca. 70–71 m AHD) and mega-lake (ca. 75 m AHD) shorelines.
(TIF)

S4 Fig Transect cross-sections 8–9 for the southwestern Lake Mungo shoreline. Inset shows the location of these transects relative to the reconstructed main (ca. 70–71 m AHD) and mega-lake (ca. 75 m AHD) shorelines.
(TIF)

S5 Fig Transect cross-sections for the southern Joulni area, transitional between the western Lake Mungo shoreline and its lunette. Transect 10 demonstrates the low elevation of the lunette at this position of the lake. Transect 11 shows the consistent elevation of beach gravels at ca. 70–71 m AHD.
(TIF)

S6 Fig Accuracy of the available elevation data sources. A. SRTM DEM showing locations where the SRTM data overestimates or underestimates elevations relative to high-resolution (1m) AHD DEMs downsampled to match the spatial resolution of the SRTM DEM, as well as the location of the N-S, W-E, and WOC beach transects. B. Elevation profiles of the SRTM and downsampled AHD DEMs along the N-S transect, illustrating the systematic overestimation of elevations in the SRTM data, as well as the extent of the water levels on the SRTM DEM if: a) using the correct, corresponding AHD elevation value, b) applying a conservative 1m correction factor, and c) applying no correction factor. C. Elevation profiles of the SRTM and downsampled AHD DEMs along the W-E transect, illustrating the systematic overestimation of elevations in the SRTM data, as well as the extent of the water levels on the SRTM DEM if: a) using the correct, corresponding AHD elevation value, b) applying a conservative 1m correction factor, and c) applying no correction factor. D. Relationship between the elevation values of the SRTM and downsampled high-resolution (1m) AHD DEMs, illustrating both the systematic bias in the SRTM data and the overall strong correlation between the SRTM and the AHD DEM data. E. Relationship between the elevation values of the high-resolution (1m) AHD DEMs and the dGPS elevation values obtained in the field, showing the strong correlation between the two datasets and the lack of notable bias.
(TIF)

S7 Fig Area used for estimating the area and volume of the Mungo mega-lake. (TIF)

S8 Fig Sedimentological characteristics of the stratigraphic units present in the central and northern parts of the lunette, from youngest (top) to oldest (bottom). (TIF)

S9 Fig Characteristics of the Red Lunette stratigraphic unit. A. In situ exposure of the Red Lunette gravel beach at 75 m AHD in the central part of the lunette, showing contacts between the underlying Upper Mungo and overlying Arumpo units. B. In situ exposure of the Red Lunette unit at 75 m AHD along the surveyed shorefront transect WOC 1. The beach gravels include non-carbonate rock gravels which indicate inflow of non-local clastic components. C. Lag surface at 73.5 m AHD in the northern part of the lunette, preserving non-local clastic components associated with the Red Lunette unit. D. Non-local clastic components collected from both in situ and lag surfaces associated with the 75 m AHD Red Lunette beach in both the northern and central parts of the lunette.
(TIF)

S10 Fig Bimodal particle size distributions, expressed as frequency percentage by volume, for the Red Lunette stratigraphic unit along the northern lunette transect. (TIF)

S11 Fig Preheat plateau test results for single aliquots of sample EVA1112. Dose response to preheat temperatures ranging between 180–280°C was measured; the results indicate no dependence of dose on preheat temperature, and therefore the preheat and cutheat temperatures of 260°C and 220°C respectively were chosen for SAR measurements of dose on all samples.
(TIF)

S12 Fig Radial plots of dose distributions for the central lunette beach samples, EVA1112-1115. Single aliquots are shown as open triangles and single grains as closed circles. The D_e_ for single aliquots is shown by the shaded grey band, and for single grains is shown as a solid black line. One exception is EVA1115, to which the finite mixture model was applied to the single grains. In this case, the different populations are shown by multiple black lines, with the thicker black line corresponding to the most likely age.
(TIF)

S13 Fig Radial plots of dose distributions for the central lunette backdune samples, EVA1116-1119. Single aliquots are shown as open triangles and single grains as closed circles. The D_e_ for single aliquots is shown by the shaded grey band, and for single grains is shown as a solid black line.
(TIF)

S14 Fig Radial plots of dose distributions for the northern lunette transect samples, EVA1255-1261. Since single grains only were measured for these samples, the radial plots show only single grain data as closed circles and the calculated D_e_ as a shaded grey band, with the exception of sample EVA1257, which was analysed using the finite mixture model. In the latter case, the different populations are shown by multiple black lines.
(TIF)

S15 Fig Radial plots of dose distributions for the channel sediment samples, EVA1265 and EVA1269. Since single grains only were measured for these samples, the radial plots show only single grain data as closed circles and the calculated D_e_ as a shaded grey band.
(TIF)

S16 Fig Radial plot summarising the results of the dose recovery test applied to sample EVA1255. (TIF)

S17 Fig Archaeological traces preserved within the RL, northern (island) transect. A. Baked sediment hearth complex, looking upslope and up-section. A ruler sits immediately to the left of the largest hearth. B.
(TIF)

S18 Fig A. Exposure of two in situ baked sediment hearths within the RL, northern (island) transect. The hearths sit at two elevations with 13 cm of sediment accretion between them (elevations shown in the insets (B) and (C)), indicating repeated occupation of the island.
(TIF)

S1 File Supplementary file containing additional information relating to methods used for lake shoreline surveys and shoreline reconstruction, sedimentological investigations, luminescence dating and archaeological observations. (DOCX)

S1 Table Summary of benching elevations representing shoreline erosion, indicating consistent elevations for the main and mega-lake shorelines. (DOCX)

S2 Table Calculated area, volume and percentage change for the main and mega-lake phases. (DOCX)

S3 Table Summary of the main characteristics and palaeoenvironmental interpretation for each late Quaternary stratigraphic unit observed within the lunette. The location, codes and ages for the OSL dating samples, including their position relative to the Red Lunette shoreline, are also given.
(DOCX)

S4 Table Summary of sedimentary characteristics for the different stratigraphic units based on thin section micromorphology, in stratigraphic order from youngest to oldest. (DOCX)

S5 Table Particle size analysis data for the Red Lunette stratigraphic unit, northern lunette transect, relative to facies. Paired OSL dating is shown, where relevant; in the cases of PSA 1–3, however, the Red Lunette unit was too thin for OSL sample collection.
(DOCX)

S6 Table Overdispersion values for OSL samples. Single aliquot results are given in plain text, single grain results in italics. Red Lunette samples are highlighted in bold type.
(DOCX)

S7 Table Results from finite mixture model analyses. (DOCX)

S8 Table Calculated concentrations of radioisotopes determined using high resolution germanium gamma spectrometry, analysed at VKTA Dresden. The gamma ray contribution to dose rates was determined from these data, using the conversion factors of Adamiec and Aitken (1998). Red Lunette data are shown in bold type.
(DOCX)

## References

[1] Bowler JM, Thorne A. Human remains from Lake Mungo: Discovery and excavation of Lake Mungo III In: Kirk R, Thorne A, editors. The origin of the Australians. Canberra: Australian Institute of Aboriginal Studies; 1976 pp. 127–138.

[2] Bowler JM, Jones R, Allen H, Thorne AG. Pleistocene human remains from Australia: A living site and human cremation from Lake Mungo, western New South Wales. World Arch.1970; 2: 39–60. 10.1080/00438243.1970.9979463 16468208

[3] Bowler JM, Johnston H, Olley JM, Prescott JR, Roberts RG, Shawcross W, et al New ages for human occupation and climatic change at Lake Mungo, Australia. Nature 2003; 421: 837–840. 12594511 10.1038/nature01383

[4] Bowler JM Willandra Lakes revisited: environmental framework for human occupation. Arch. in Oceania 1998; 33: 120–155.

[5] Bowler JM, Gillespie R, Johnston H, Boljkovac K. Wind v water: Glacial maximum records from the Willandra Lakes In: Haberle S, David B, editors. Peopled landscapes: archaeological and biogeographic approaches to landscapes. Canberra: The Australian National University; 2012 pp. 271–296.

[6] Fitzsimmons KE, Stern N, Murray-Wallace CV. Depositional history and archaeology of the central Lake Mungo lunette, Willandra Lakes, southeast Australia. Journal of Archaeological Science 2014; 41: 349–364.

[7] Stern N, Tumney J, Fitzsimmons KE, Kajewski P. Strategies for investigating human responses to changes in landscape and climate at Lake Mungo in the Willandra Lakes, southeast Australia In: Archaeology in environment and technology: Intersections and Transformations. Frankel D, Webb J, Lawrence S, editors. New York: Routledge; 2013 pp. 31–50.

[8] Bowler JM Aridity in Australia: Age, origins and expression in aeolian landforms and sediments. Earth Sci. Rev. 1976; 12: 279–310.

[9] Bowler JM. Spatial variability and hydrologic evolution of Australian lake basins: analogue for Pleistocene hydrologic change and evaporite formation. Palaeogeog. Palaeoclim. Palaeoecol. 1986; 54: 21–41.

[10] Kemp J. Flood channel morphology of a quiet river, the Lachlan downstream from Cowra, southeastern Australia. Geomorph. 2004; 60: 171–190.

[11] Kemp J, Spooner NA. Evidence for regionally wet conditions before the LGM in southeast Australia: OSL ages from a large palaeochannel in the Lachlan Valley. J. Quat. Sci. 2007; 22: 423–427.

[12] Kemp J, Rhodes EJ. Episodic fluvial activity of inland rivers in southeastern Australia: Palaeochannel systems and terraces of the Lachlan River Quat. Sci. Rev. 2010; 29: 732–752.

[13] Petherick L, Bostock H, Cohen TJ, Fitzsimmons KE, Tibby J, Moss P, et al Climatic records over the past 35 ka from temperate Australia–a synthesis from the OZ-INTIMATE workgroup. Quat. Sci. Rev. 2013; 74: 58–77.

[14] Reeves JM, Barrows TT, Cohen TJ, Kiem AS, Bostock H, Fitzsimmons KE, et al Global climate variability recorded in marine and terrestrial archives in the Australian region over the last 35 ka: an OZ-INTIMATE compilation. Quat. Sci. Rev. 2013; 74: 21–34.

[15] Bowler JM, Magee JW. Geomorphology of the Mallee region in semi-arid northern Victoria and western New South Wales. Proc. Roy. Soc. Victoria 1978; 90: 5–25.

[16] Lomax J, Hilgers A, Radtke U. Palaeoenvironmental change recorded in the palaeodunefields of the western Murray Basin, South Australia—New data from single grain OSL-dating. Quat. Sci. Rev. 2011; 30: 723–736.

[17] Fitzsimmons KE, Cohen TJ, Hesse PP, Jansen J, Nanson GC, May JH, et al Late Quaternary palaeoenvironmental change in the Australian drylands: a synthesis. Quat. Sci. Rev. 2013; 74: 78–96.

[18] Douglas K. Land systems and stratigraphy of Lake Mulurulu: examination of Quaternary palaeoenvironments. BSc(Hons) thesis. Melbourne: University of Melbourne; 1996.

[19] Clark D. A seismic source zone model based on neotectonics data In: Earthquake Engineering in Australia. Canberra: Australian Earthquake Engineering Society; 2006 pp. 69–76.

[20] Kellett J. The Ivanhoe Block—its structure, hydrogeology and effect on groundwaters of the Riverine Plain of New South Wales. BMR J. Geol. Geophys. 1989; 11: 333–353.

[21] Lawrie KC, Brodie RS, Dillon P, Tan KP, Gibson D, Magee JW, et al Broken Hill Managed Aquifer Recharge (BHMAR) Project: Assessment of Conjunctive Water Supply Options to Enhance the Drought Security of Broken Hill, Regional Communities and Industries: Summary Report. Canberra: Geoscience Australia; 2012.

[22] Bourne JA, Twidale CR. Lineaments and cryptostructural effects In: Crustal structure and mineral deposits–E. S. T. O’Driscoll’s contributions to mineral exploration. Bourne JA, editor. Sydney: Rosenberg; 2007 pp. 153–164.

[23] Long K, Stern N, Williams IS, Kinsley L, Wood R, Sporcic K, et al Fish otolith geochemistry, environmental conditions and human occupation at Lake Mungo, Australia. Quat. Sci. Rev. 2014; 88: 82–95.

[24] Radczewski OE. Eolian deposits on marine sediments, in Recent Marine Sediments: A Symposium In: Trask PD, editor. Oklahoma: The American Association of Petroleum Geologists; 1939 pp. 496–502.

[25] Page KJ, Nanson GC, Price D. Chronology of Murrumbidgee river palaeochannels on the Riverine Plain, southeastern Australia. J. Quat. Sci. 1996; 11: 311–326.

[26] Page KJ, Dare-Edwards AJ, Owens JW, Frazier PS, Kellett J, Price DM. TL chronology and stratigraphy of riverine source bordering sand dunes near Wagga Wagga, New South Wales, Australia. Quat. Int. 2001; 83–85: 187–193.

[27] Nanson GC, Cohen TJ, Doyle CJ, Price DM. Alluvial evidence of Late-Quaternary climate and flow-regime changes on the coastal rivers of New South Wales, Australia In: Gregory KJ, Benito G, editors. Palaeohydrology: Understanding Global Change. Chichester: Wiley; 2003 pp. 233–258.

[28] Page KJ, Dare-Edwards AJ, Nanson GC, Price DM. Late quaternary evolution of Lake Urana, New South Wales, Australia. J. Quat. Sci. 1994; 9: 47–57.

[29] Barrows TT, Stone JO, Fifield LK, Creswell RG. Late Pleistocene glaciation of the Kosciuszko Massif, Snowy Mountains, Australia. Quat. Res. 2001; 55: 179–189.

[30] Barrows TT, Stone JO, Fifield LK. Exposure ages for Pleistocene periglacial deposits in Australia. Quat. Sci. Rev. 2004; 23: 697–708.

[31] Costin AB, Polach HA. Age and significance of slope deposits, Black Mountain, Canberra. Aust. J. Soil Res. 1973; 11: 13–25.

[32] Quigley MC, Cupper ML, Sandiford M. Quaternary faults of south-central Australia: Palaeoseismicity, slip rates and origin. Aust. J. Earth Sci. 2006; 53: 285–301.

[33] McPherson AA, Clark D, Cupper M, Collins CDN, Nelson G. The Cadell Fault: a record of long-term fault behaviour in south-eastern Australia. In: Australian Regolith and Clays Conference. Mildura: Australian Regolith Geoscientists Association; 2012. pp. 7–16.

[34] Stone T. Late Quaternary rivers and lakes of the Cadell Tilt Block region, Murray Basin, southeastern Australia. PhD thesis. Melbourne: University of Melbourne; 2006.

[35] Petherick L, McGowan H, Moss P. Climate variability during the Last Glacial Maximum in eastern Australia: evidence of two stadials? J. Quat. Sci. 2008; 23: 787–802.

[36] Hesse PP. The record of continental dust from Australia in Tasman Sea sediments. Quat. Sci. Rev. 1994; 13: 257–272.

[37] Hesse PP, Magee JW, van der Kaars S. Late Quaternary climates of the Australian arid zone: A review. Quat. Int. 2004; 118–119: 87–102.

[38] Webb S, Cupper ML, Robins R. Pleistocene human footprints from the Willandra Lakes, southeastern Australia. J. Human Evol. 2006; 50: 405–413. 16343597 10.1016/j.jhevol.2005.10.002

[39] Holdaway S, Douglass M. A Twenty-First Century Archaeology of Stone Artifacts. J. Arch. Meth. Theory 2012; 19: 101–131.

[40] Mountford C. An unrecorded method of manufacturing wooden implements by simple stone tools. Trans. R. Soc. South Aust. 1941; 65: 312–316.

[41] Tindale N. The Pitjandjara In: Bichierri M, editor. Hunters and Gatherers Today. New York: Holt, Rinehart and Winston; 1972 pp. 217–268.

[42] Gould RA. The anthropology of human residues. Amer. Anth. 1978; 80: 815–835.

[43] O’Connell JF, Allen J, Hawkes K. Pleistocene Sahul and the origins of seafaring In: Anderson A, Barrett JH, Boyle KV, editors. The global origins and development of seafaring. Cambridge: University of Cambridge; 2010 pp. 57–68.

[44] O’Connor S, Chappell J. Colonisation and coastal subsistence in Australia and Papua New Guinea: different timing, different modes In: Sand C, editor. Pacific Archaeology: assessments and prospects. Noumea: Département Archéologie, Service des Musées et du Patrimoine de Nouvelle-Calédonie; 2003 pp. 17–32.

[45] O’Connor S, Veth P. The world’s first mariners: savannah dwellers in an island continent In: O’Connor S, Veth P, editors. East of Wallace’s Line: Studies of past and present maritime cultures of the Indo-Pacific region. Rotterdam: Balkema; 2000 pp. 99–138.

[46] Balme J. Of boats and string: The maritime colonisation of Australia. Quat. Int. 2013; 285: 68–75.

[47] Jarvis A, Reuter HI, Nelson A, Guevara E. Hole-filled SRTM for the globe Version 4, CGIAR-SXI SRTM 90m database; 2009. Available: http://srtm.csi.cgiar.org

[48] Fitzsimmons KE, Magee JW, Amos K. Characterisation of aeolian sediments from the Strzelecki and Tirari Deserts, Australia: implications for reconstructing palaeoenvironmental conditions. Sed. Geol. 2009; 218: 61–73.

[49] Bøtter-Jensen L. Luminescence techniques: instrumentation and methods. Rad. Meas. 1997; 27: 749–768.

[50] Bøtter-Jensen L, Bulur E, Duller GAT, Murray AS. Advances in luminescence instrument systems. Rad. Meas. 2000; 32: 523–528.

[51] Murray AS, Wintle AG. Luminescence dating of quartz using an improved single-aliquot regenerative-dose protocol. Rad. Meas. 2000; 32: 57–73.

[52] Murray AS, Wintle AG. The single aliquot regenerative dose protocol: potential for improvements in reliability. Rad. Meas. 2003; 37: 377–381.

[53] Galbraith RF, Roberts RG, Laslett GM, Yoshida H, Olley JM. Optical dating of single and multiple grains of quartz from Jinmium rock shelter, northern Australia. Part 1, Experimental design and statistical models. Archaeom. 1999; 41: 339–364.

[54] Galbraith RF, Green PF (1990) Estimating the component ages in a finite mixture. Nuclear Tracks and Rad. Meas. 1990; 17: 197–206.

[55] Adamiec G, Aitken M. Dose-rate conversion factors: update. Ancient TL 1998; 16: 37–50.

[56] Mejdahl V. Thermoluminescence dating: beta-dose attenuation in quartz grains. Archaeometry 1979; 21: 61–72.

[57] Prescott JR, Hutton JT. Cosmic ray contributions to dose rates for luminescence and ESR dating: Large depths and long term variations. Rad. Meas. 1994; 23(2–3): 497–500.

