# Supplementary figures and images for: The Mungo Mega-Lake Event, Semi-Arid Australia: Non-Linear Descent into the Last Ice Age, Implications for Human Behaviour
Source: PLoS One. 2015 Jun 17;10(6):e0127008. doi: 10.1371/journal.pone.0127008 (PMC4470511; doi:10.1371/journal.pone.0127008)

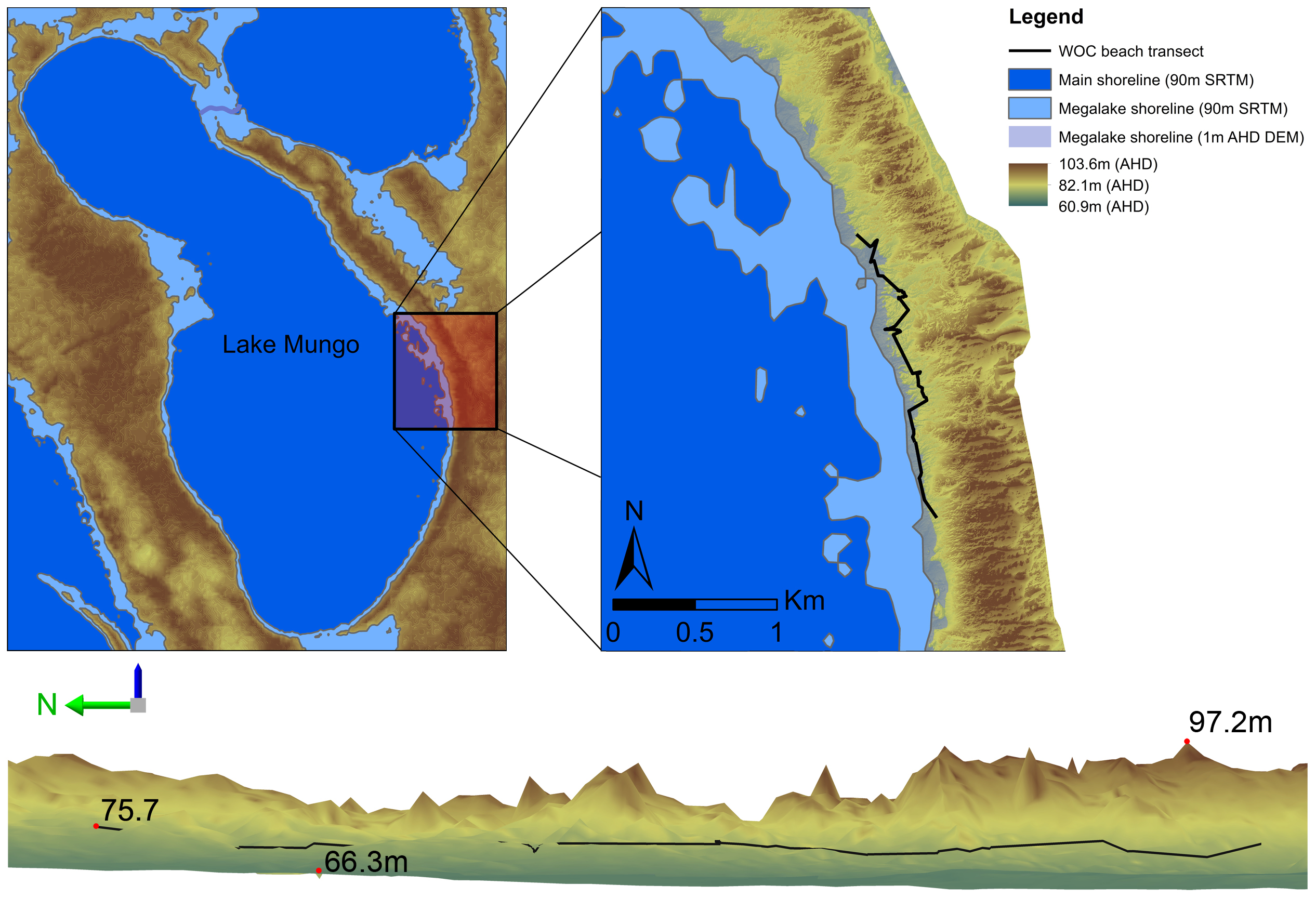

Supplement: S1 Fig — Clockwise from top left: Location of the transect within the Lake Mungo lunette; transect from ground truthing, based on observation of shoreline features; lateral view of consistency of shoreline elevation, projected onto the digital elevation model generated from the aerial photos collected from the central portion of the lunette. Note that the dGPS data and aerial photo data are more accurate than the shoreline reconstruction based on the SRTM data, which accounts for the divergence between the directly surveyed shoreline and that shown in the map top right. (TIF) [file pone.0127008.s001.tif]

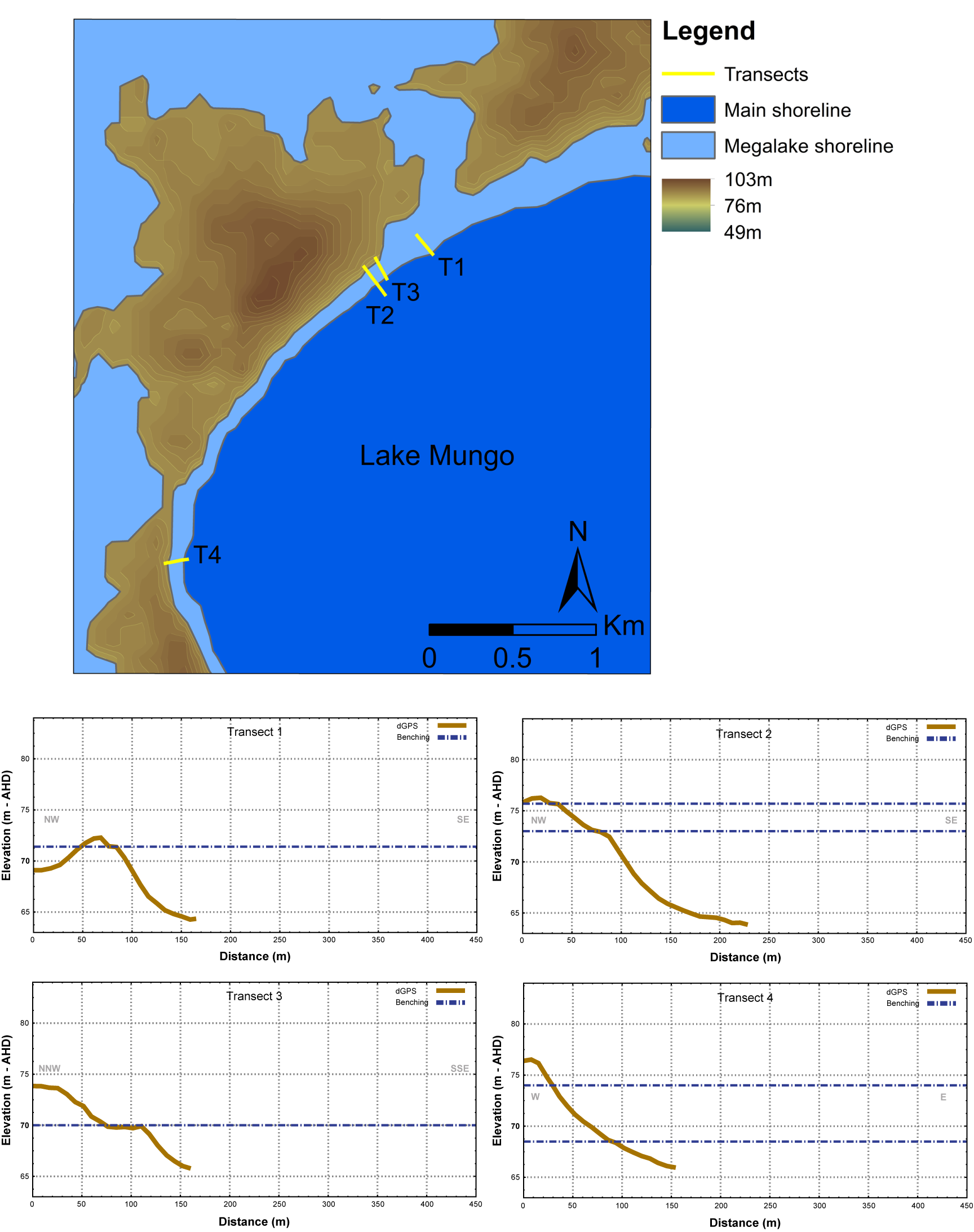

Supplement: S2 Fig — Inset shows the location of these transects relative to the reconstructed main (ca. 70–71 m AHD) and mega-lake (ca. 75 m AHD) shorelines. (TIF) [file pone.0127008.s002.tif]

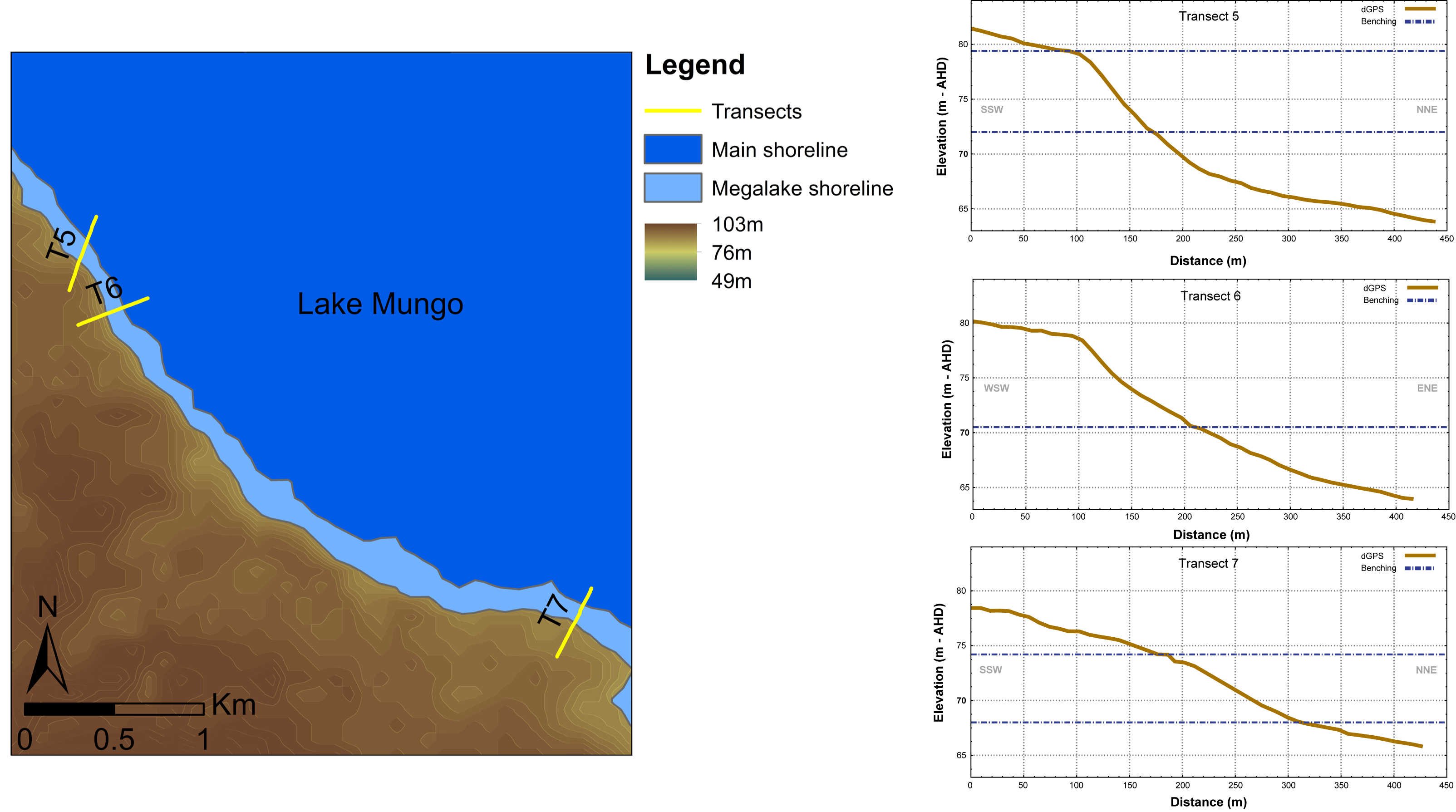

Supplement: S3 Fig — Inset shows the location of these transects relative to the reconstructed main (ca. 70–71 m AHD) and mega-lake (ca. 75 m AHD) shorelines. (TIF) [file pone.0127008.s003.tif]

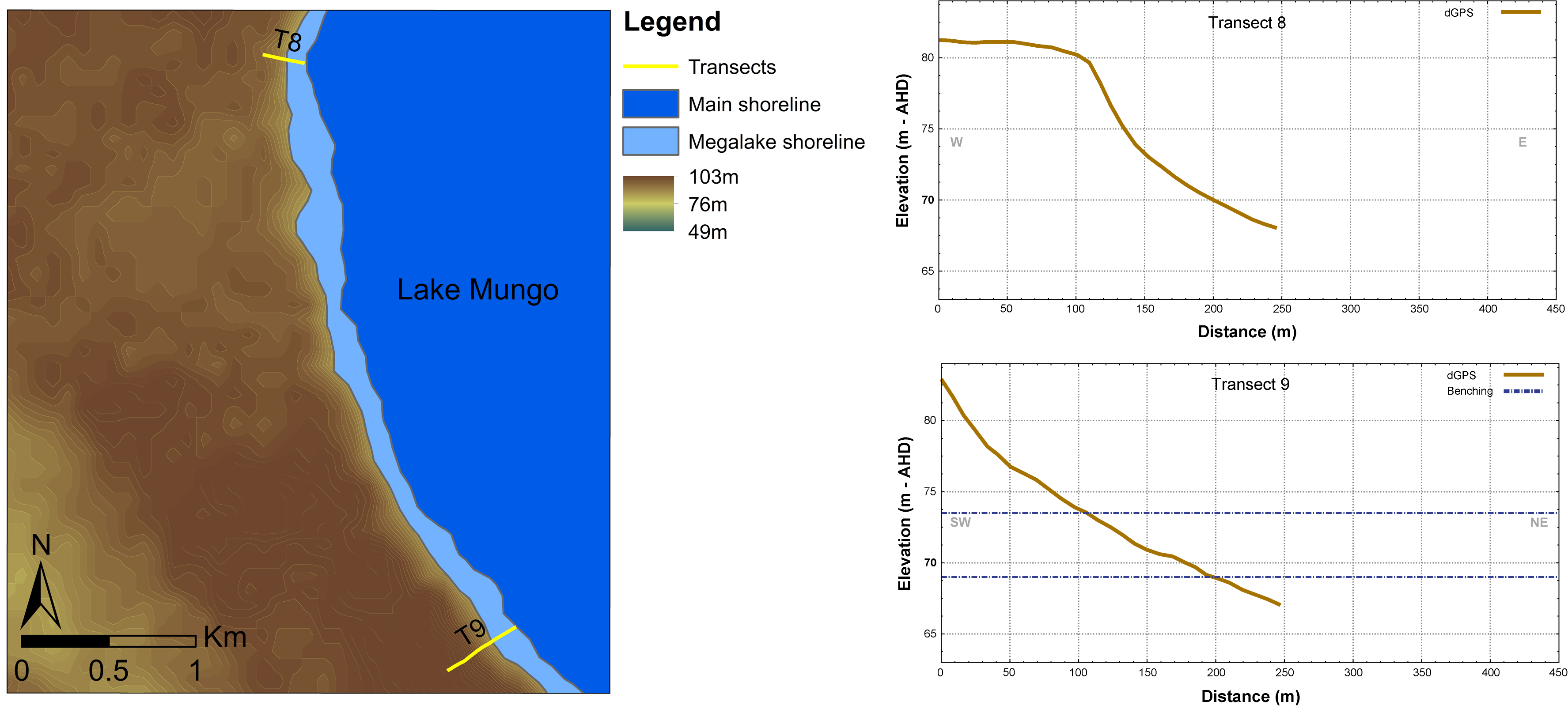

Supplement: S4 Fig — Inset shows the location of these transects relative to the reconstructed main (ca. 70–71 m AHD) and mega-lake (ca. 75 m AHD) shorelines. (TIF) [file pone.0127008.s004.tif]

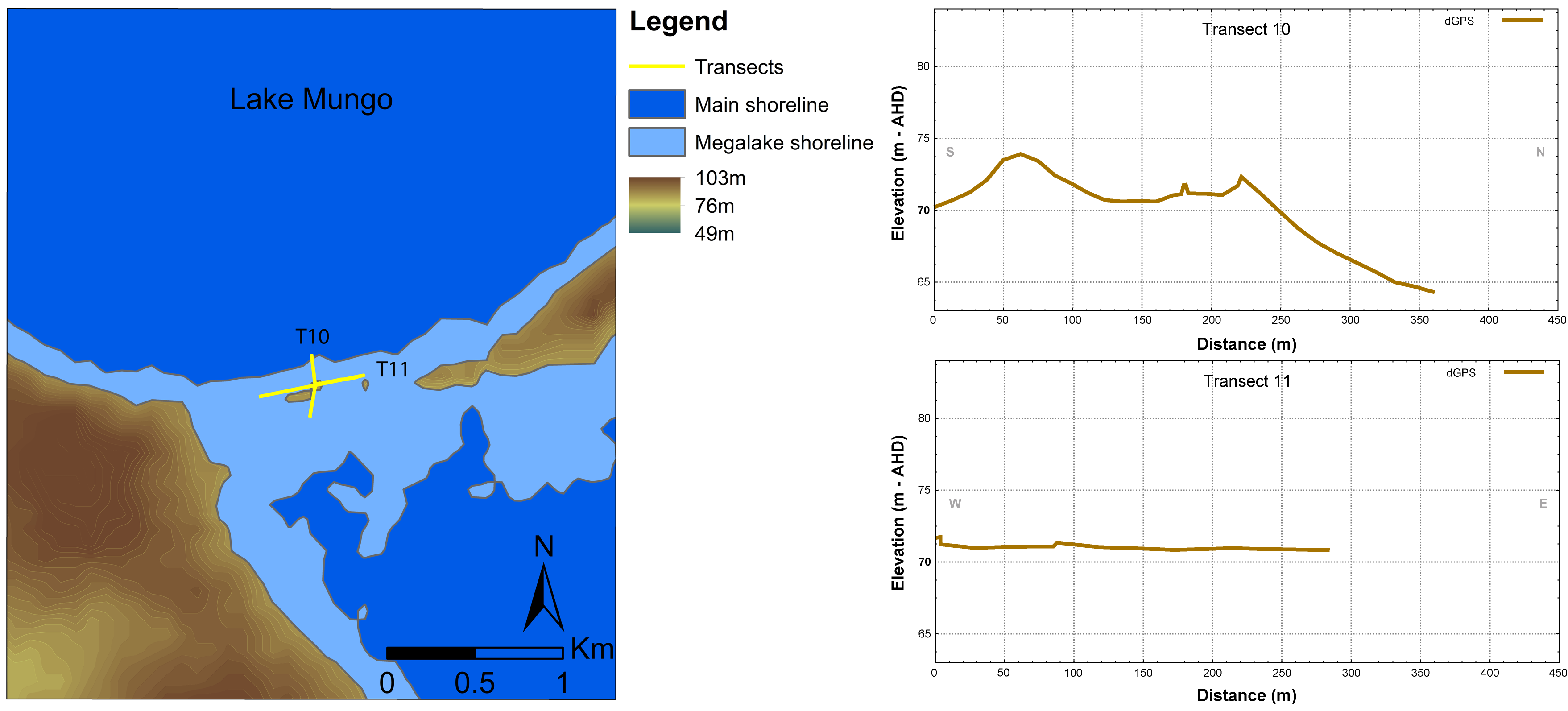

Supplement: S5 Fig — Transect 10 demonstrates the low elevation of the lunette at this position of the lake. Transect 11 shows the consistent elevation of beach gravels at ca. 70–71 m AHD. (TIF) [file pone.0127008.s005.tif]

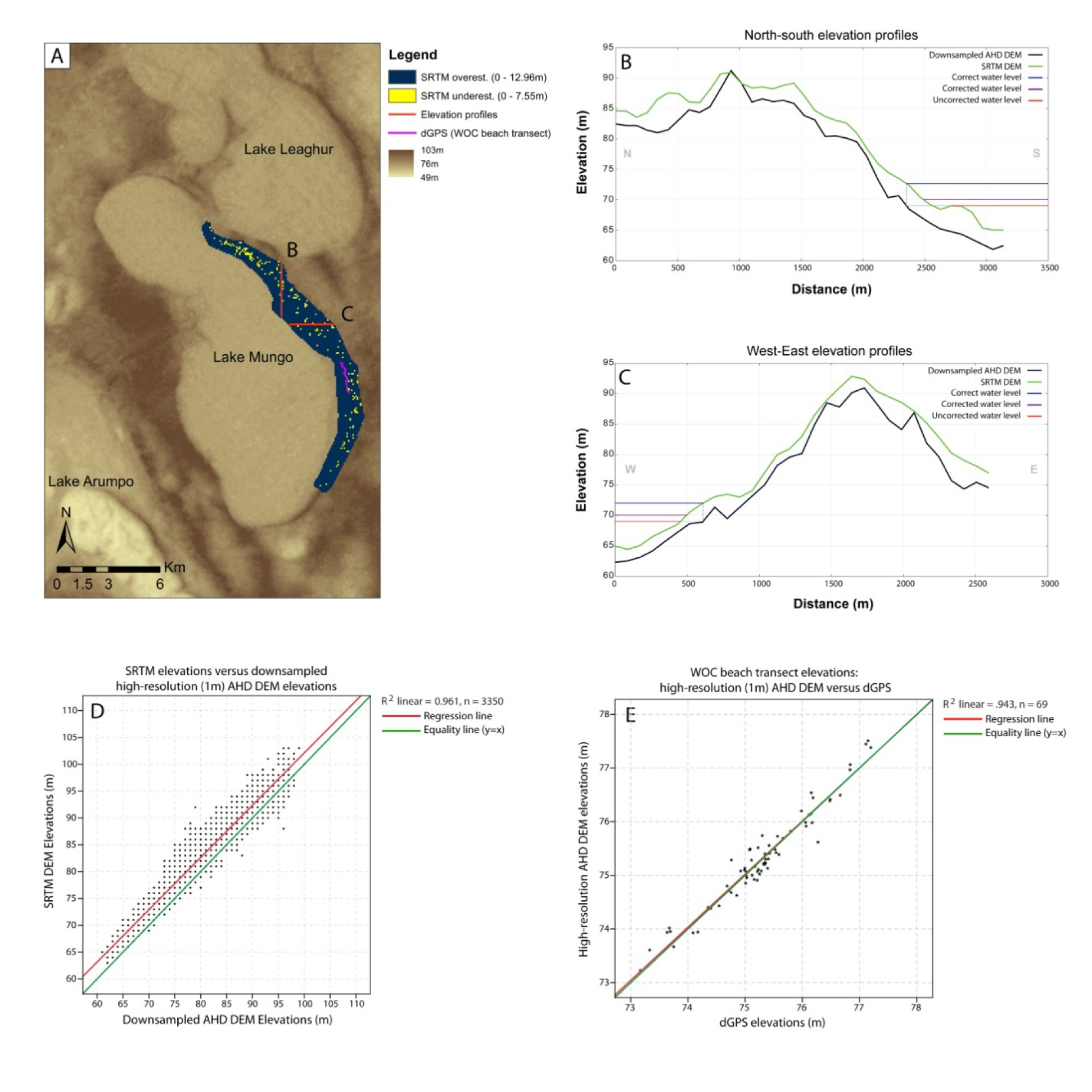

Supplement: S6 Fig — A. SRTM DEM showing locations where the SRTM data overestimates or underestimates elevations relative to high-resolution (1m) AHD DEMs downsampled to match the spatial resolution of the SRTM DEM, as well as the location of the N-S, W-E, and WOC beach transects. B. Elevation profiles of the SRTM and downsampled AHD DEMs along the N-S transect, illustrating the systematic overestimation of elevations in the SRTM data, as well as the extent of the water levels on the SRTM DEM if: a) using the correct, corresponding AHD elevation value, b) applying a conservative 1m correction factor, and c) applying no correction factor. C. Elevation profiles of the SRTM and downsampled AHD DEMs along the W-E transect, illustrating the systematic overestimation of elevations in the SRTM data, as well as the extent of the water levels on the SRTM DEM if: a) using the correct, corresponding AHD elevation value, b) applying a conservative 1m correction factor, and c) applying no correction factor. D. Relationship between the elevation values of the SRTM and downsampled high-resolution (1m) AHD DEMs, illustrating both the systematic bias in the SRTM data and the overall strong correlation between the SRTM and the AHD DEM data. E. Relationship between the elevation values of the high-resolution (1m) AHD DEMs and the dGPS elevation values obtained in the field, showing the strong correlation between the two datasets and the lack of notable bias. (TIF) [file pone.0127008.s006.tif]

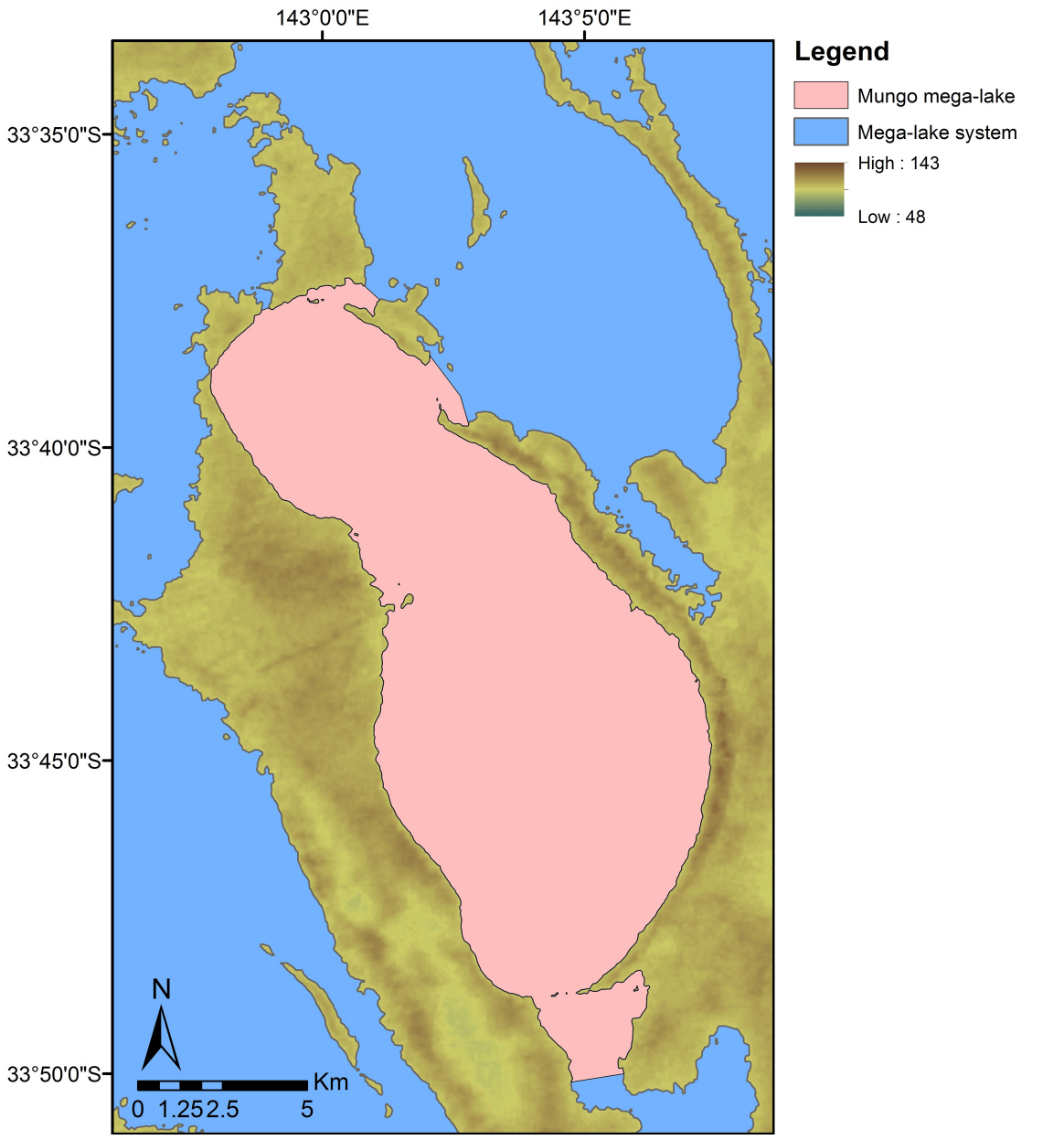

Supplement: S7 Fig — (TIF) [file pone.0127008.s007.tif]

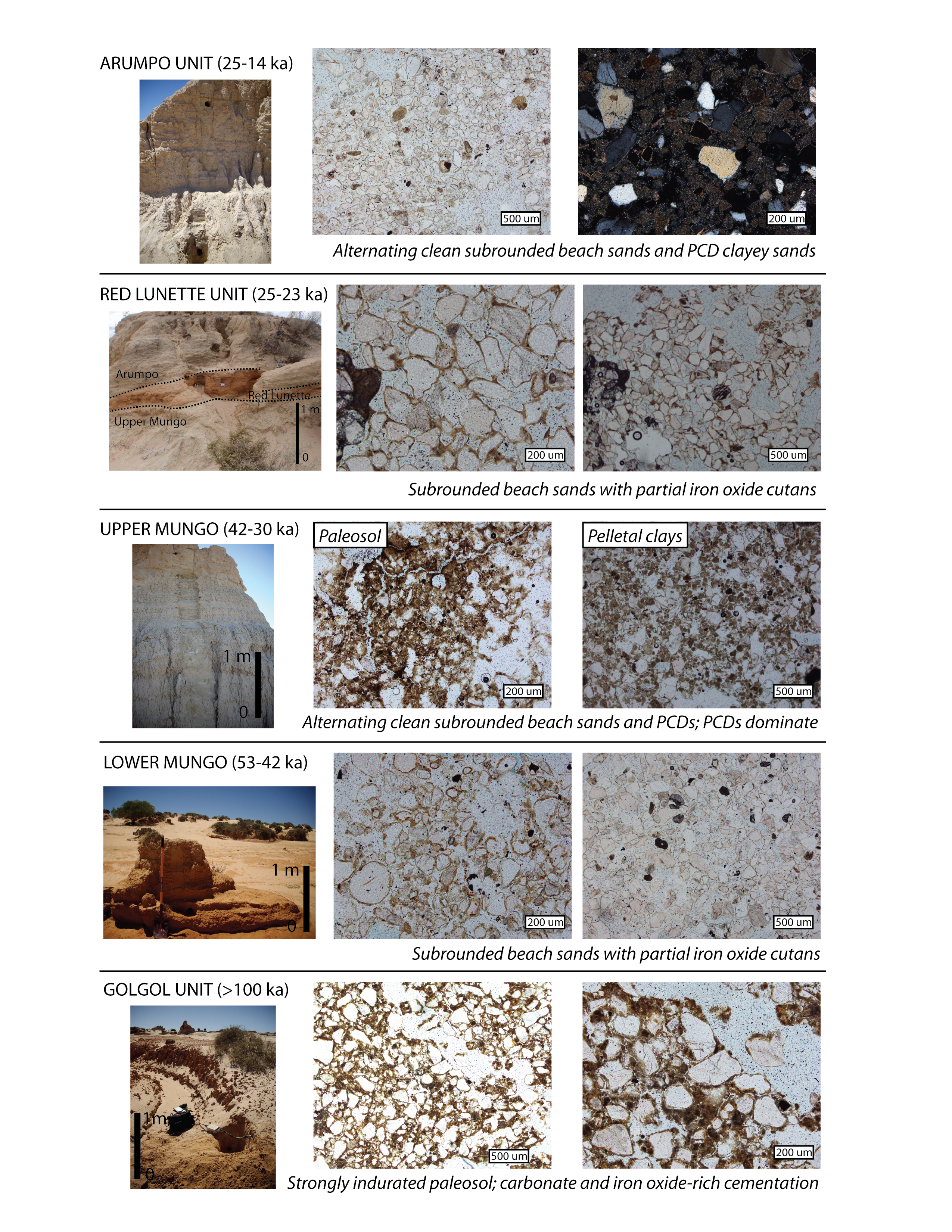

Supplement: S8 Fig — (TIF) [file pone.0127008.s008.tif]

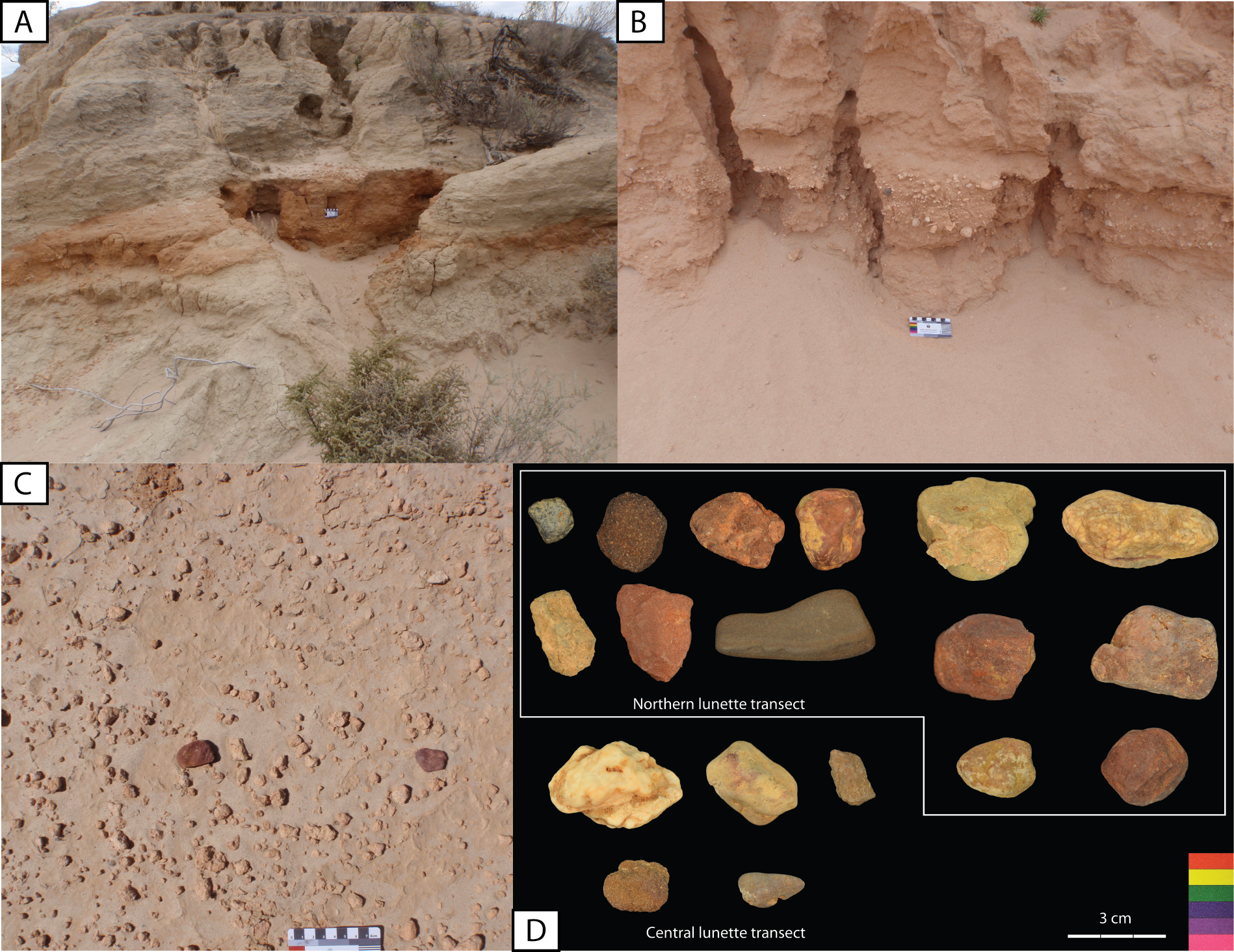

Supplement: S9 Fig — A. In situ exposure of the Red Lunette gravel beach at 75 m AHD in the central part of the lunette, showing contacts between the underlying Upper Mungo and overlying Arumpo units. B. In situ exposure of the Red Lunette unit at 75 m AHD along the surveyed shorefront transect WOC 1. The beach gravels include non-carbonate rock gravels which indicate inflow of non-local clastic components. C. Lag surface at 73.5 m AHD in the northern part of the lunette, preserving non-local clastic components associated with the Red Lunette unit. D. Non-local clastic components collected from both in situ and lag surfaces associated with the 75 m AHD Red Lunette beach in both the northern and central parts of the lunette. (TIF) [file pone.0127008.s009.tif]

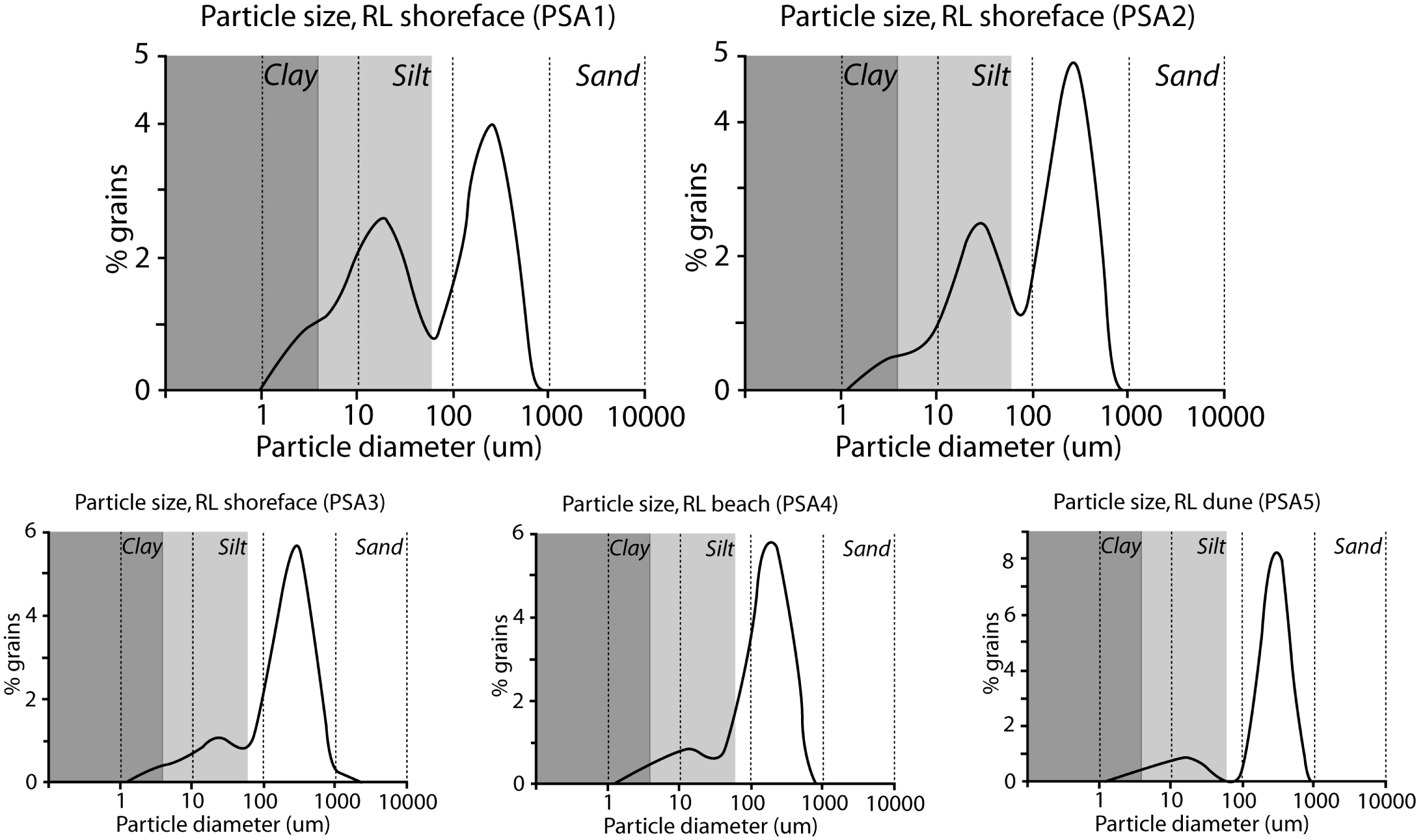

Supplement: S10 Fig — (TIF) [file pone.0127008.s010.tif]

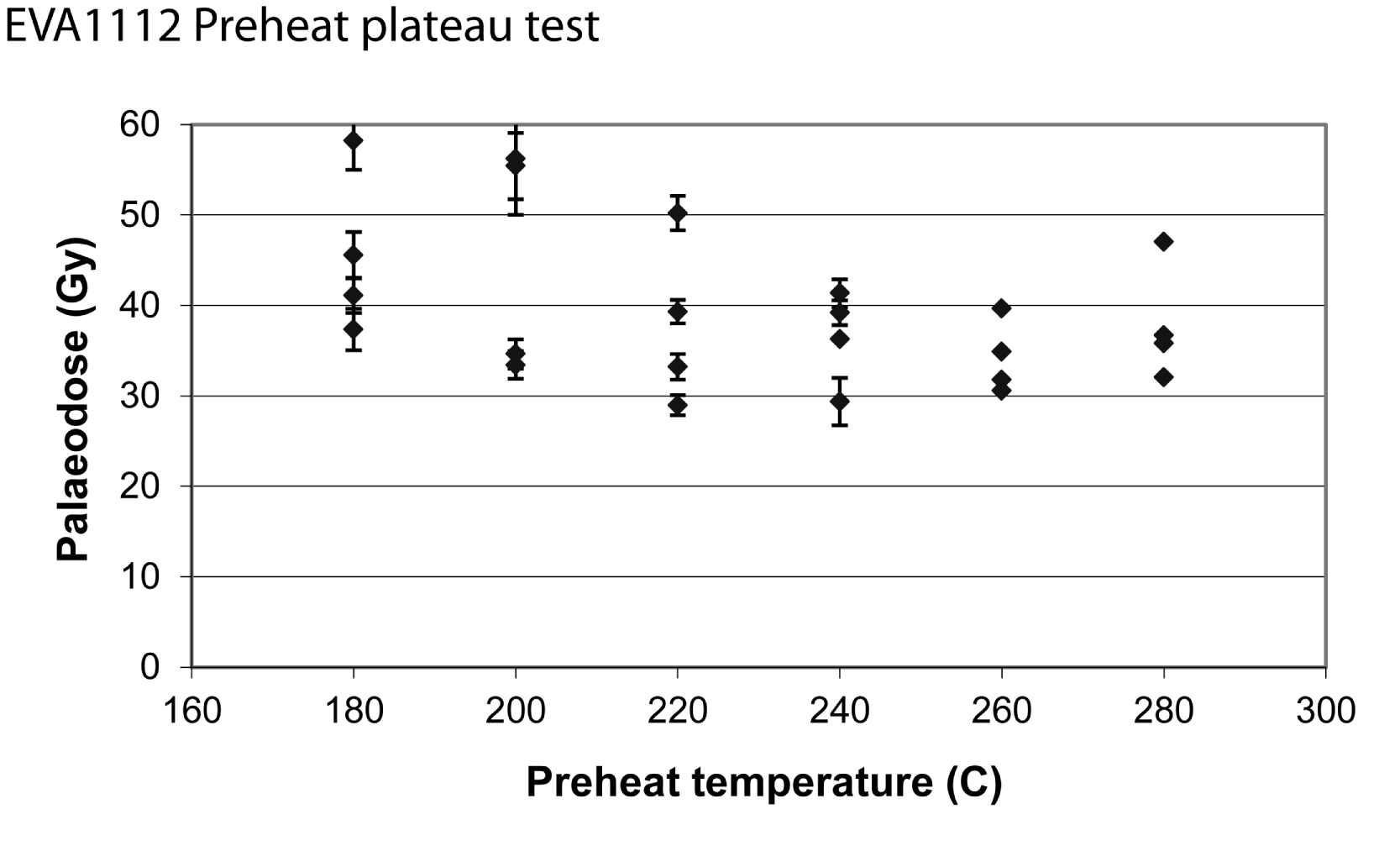

Supplement: S11 Fig — Dose response to preheat temperatures ranging between 180–280°C was measured; the results indicate no dependence of dose on preheat temperature, and therefore the preheat and cutheat temperatures of 260°C and 220°C respectively were chosen for SAR measurements of dose on all samples. (TIF) [file pone.0127008.s011.tif]

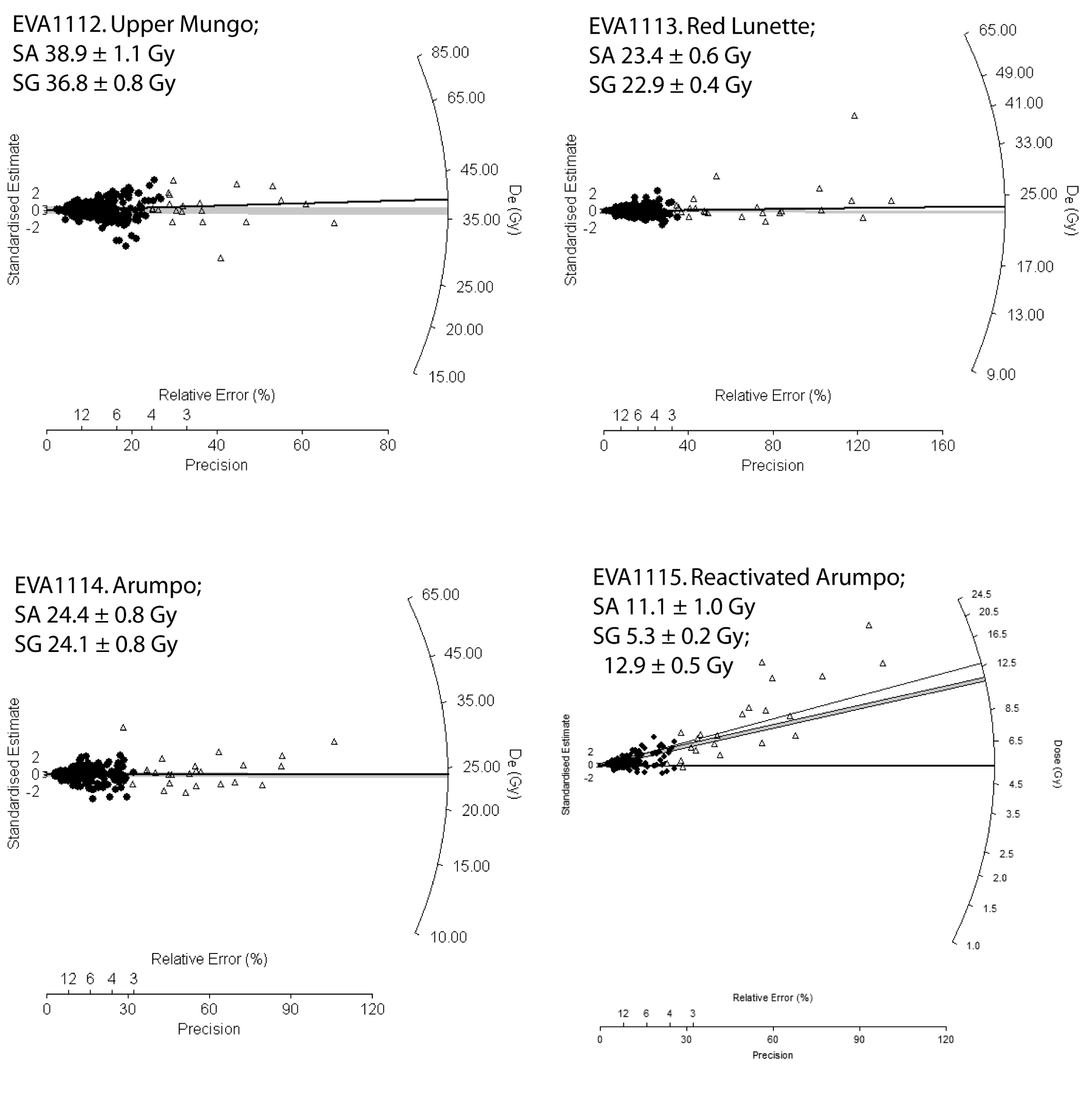

Supplement: S12 Fig — Single aliquots are shown as open triangles and single grains as closed circles. The De for single aliquots is shown by the shaded grey band, and for single grains is shown as a solid black line. One exception is EVA1115, to which the finite mixture model was applied to the single grains. In this case, the different populations are shown by multiple black lines, with the thicker black line corresponding to the most likely age. (TIF) [file pone.0127008.s012.tif]

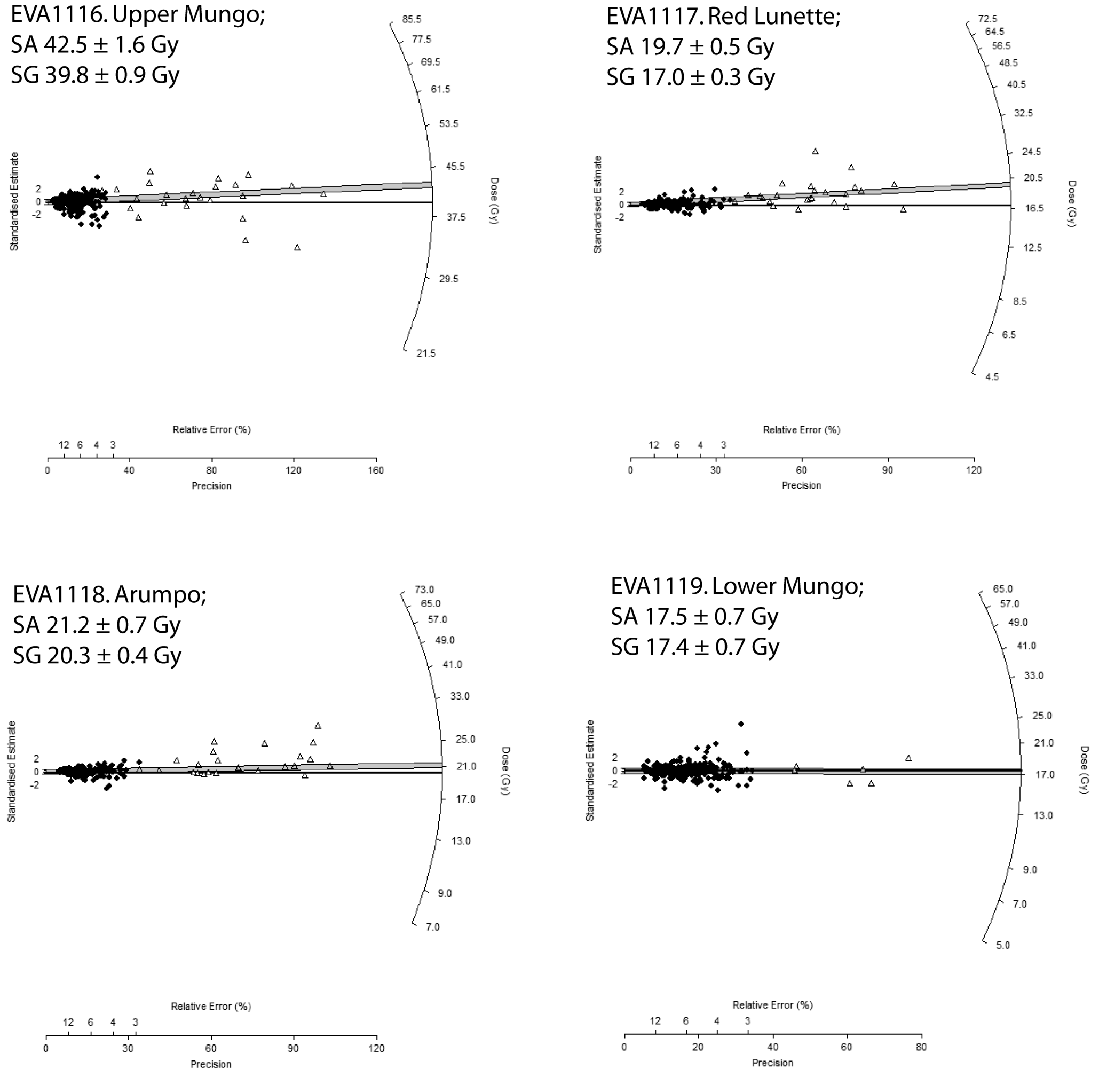

Supplement: S13 Fig — Single aliquots are shown as open triangles and single grains as closed circles. The De for single aliquots is shown by the shaded grey band, and for single grains is shown as a solid black line. (TIF) [file pone.0127008.s013.tif]

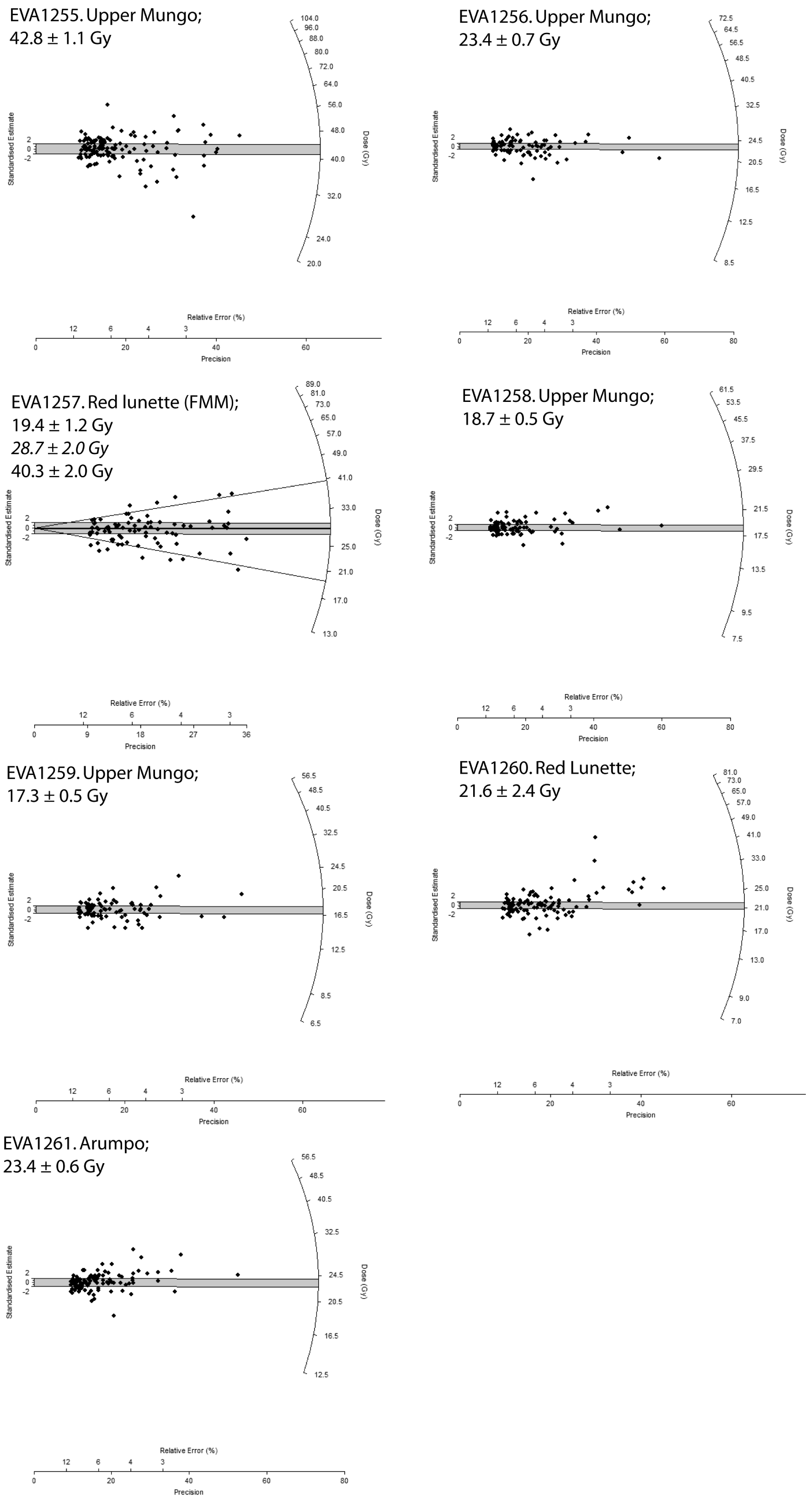

Supplement: S14 Fig — Since single grains only were measured for these samples, the radial plots show only single grain data as closed circles and the calculated De as a shaded grey band, with the exception of sample EVA1257, which was analysed using the finite mixture model. In the latter case, the different populations are shown by multiple black lines. (TIF) [file pone.0127008.s014.tif]

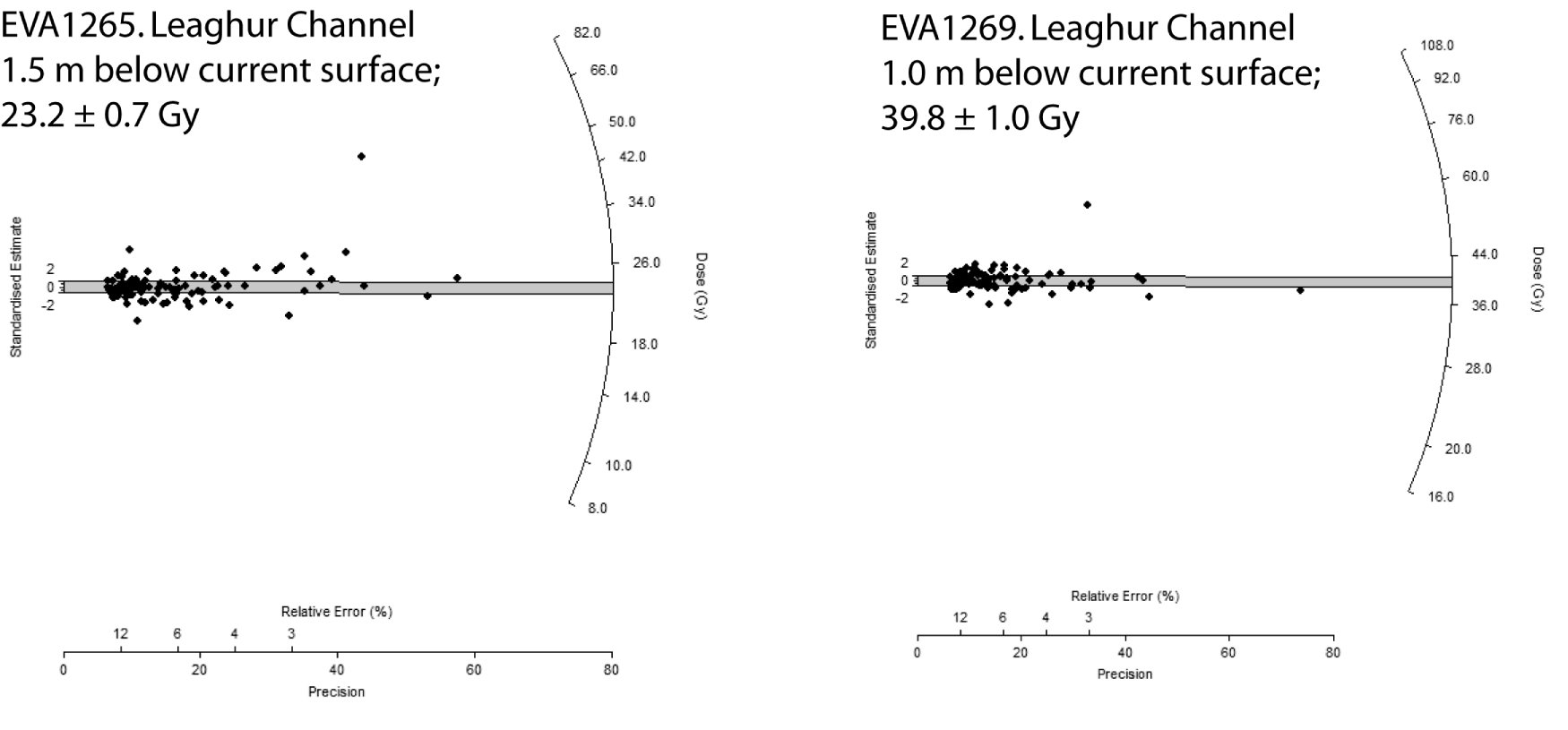

Supplement: S15 Fig — Since single grains only were measured for these samples, the radial plots show only single grain data as closed circles and the calculated De as a shaded grey band. (TIF) [file pone.0127008.s015.tif]

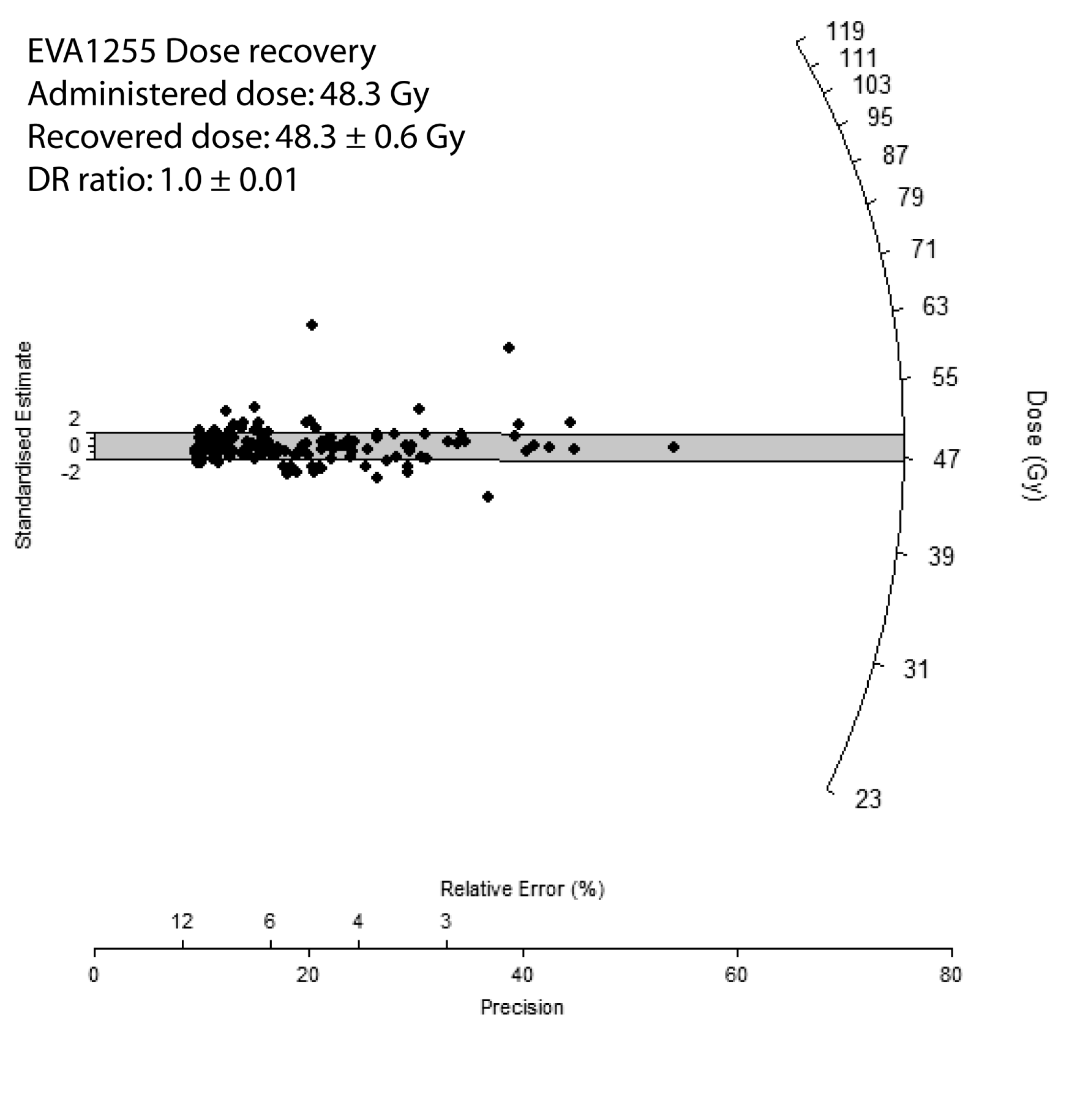

Supplement: S16 Fig — (TIF) [file pone.0127008.s016.tif]

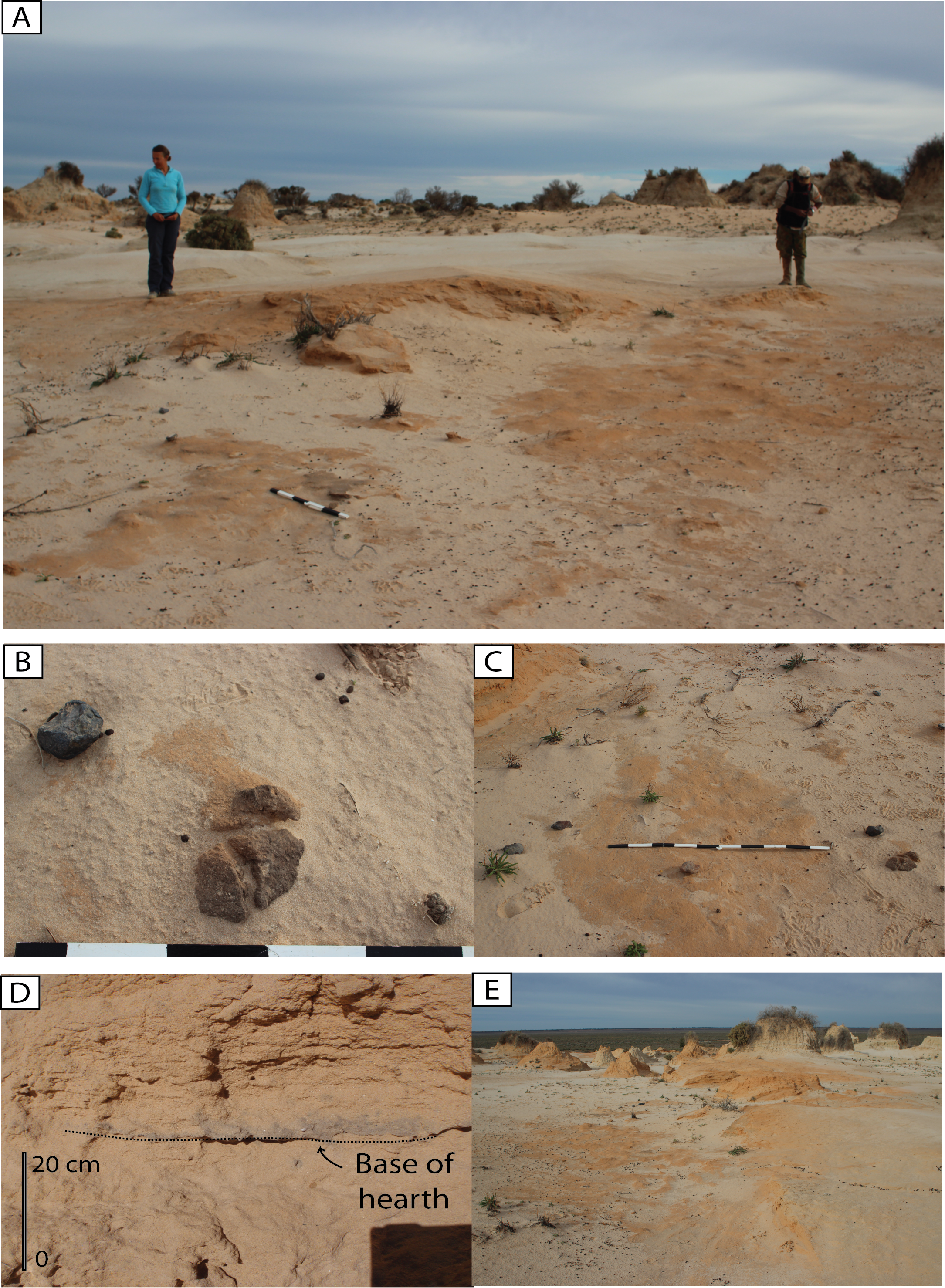

Supplement: S17 Fig — A. Baked sediment hearth complex, looking upslope and up-section. A ruler sits immediately to the left of the largest hearth. B. (TIF) [file pone.0127008.s017.tif]

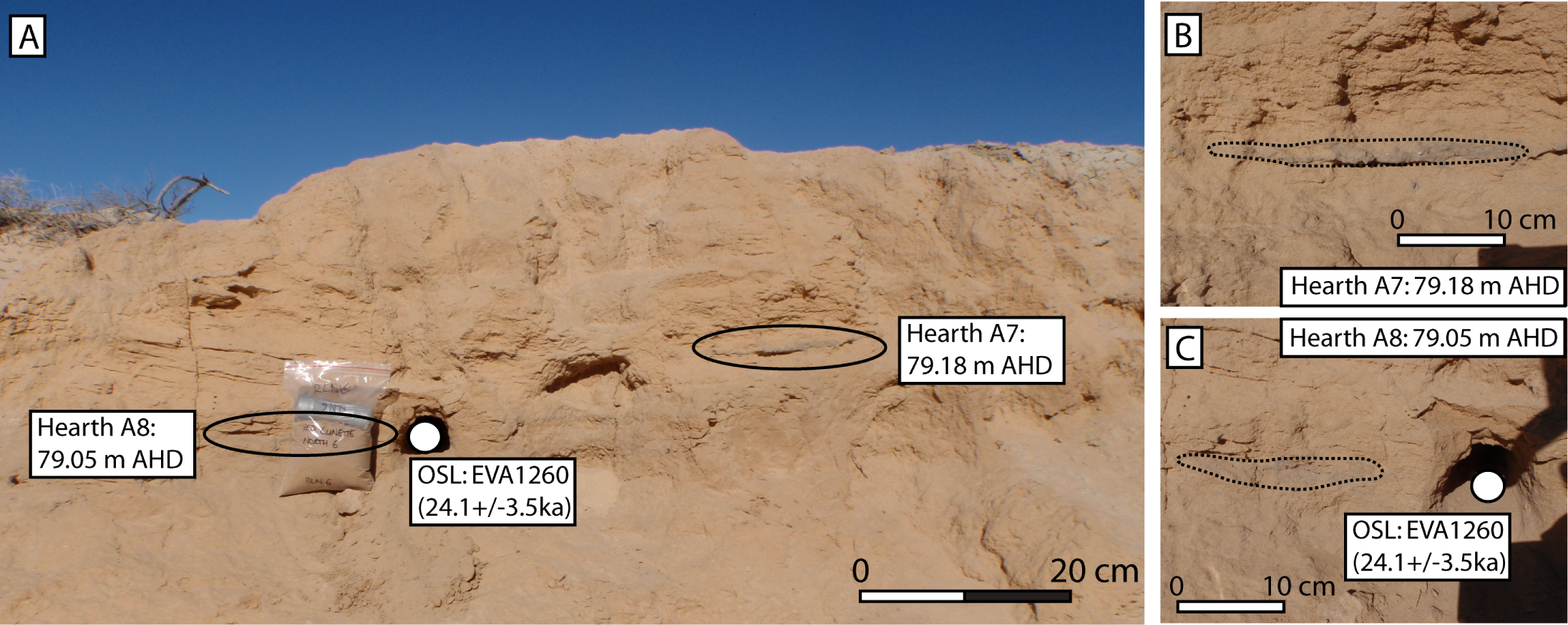

Supplement: S18 Fig — The hearths sit at two elevations with 13 cm of sediment accretion between them (elevations shown in the insets (B) and (C)), indicating repeated occupation of the island. (TIF) [file pone.0127008.s018.tif]
