# Supplementary material for: The Mungo Mega-Lake Event, Semi-Arid Australia: Non-Linear Descent into the Last Ice Age, Implications for Human Behaviour
Source: PLoS One. 2015 Jun 17;10(6):e0127008. doi: 10.1371/journal.pone.0127008 (PMC4470511; doi:10.1371/journal.pone.0127008)
Supplement: S1 File — (DOCX) [file pone.0127008.s019.docx]

**Supporting information**

**Lake shoreline surveys**

Ground truthing of the mega-lake shoreline was undertaken using differential GPS (dGPS) surveys georectified to Geodetic Datum of Australia (GDA) 1994, MGA zone 54, using the Australian Height Datum (AHD). The consistency of elevation of the Red Lunette mega-lake shoreline, relative to the main shoreline corresponding to the Mungo and Arumpo phases, was confirmed by dGPS surveying of a 2.54 km transect (the WOC beach transect) along the central part of the lunette (Figure S1), in which shoreline features such as beach gravels were identified at all available exposures.

**Figure S1. Confirmation of the elevation consistency of the Red Lunette mega-lake shoreline at ca. 75 m AHD, based on dGPS measurements taken during ground truthing. Clockwise from top left: Location of the transect within the Lake Mungo lunette; transect from ground truthing, based on observation of shoreline features; lateral view of consistency of shoreline elevation, projected onto the digital elevation model generated from the aerial photos collected from the central portion of the lunette. Note that the dGPS data and aerial photo data are more accurate than the shoreline reconstruction based on the SRTM data, which accounts for the divergence between the directly surveyed shoreline and that shown in the map top right.**

Topographic transects of the western Lake Mungo shoreline were also undertaken at 9 locations using dGPS to identify potential benching during high lake phases. Benching was not observed at all locations, most likely due to erosion of the shorelines and redeposition of sediment towards the base of the slopes. A summary of observations of benching is given in Table S1; cross sections of topography based on dGPS surveying are summarised in Figures S2-4.

**Table S1. Summary of benching elevations representing shoreline erosion, indicating consistent elevations for the main and mega-lake shorelines.**

**Figure S2. Transect cross-sections 1-4 for the northwestern Lake Mungo shoreline. Inset shows the location of these transects relative to the reconstructed main (ca. 70-71 m AHD) and mega-lake (ca. 75 m AHD) shorelines.**

**Figure S3. Transect cross-sections 5-7 for the western Lake Mungo shoreline. Inset shows the location of these transects relative to the reconstructed main (ca. 70-71 m AHD) and mega-lake (ca. 75 m AHD) shorelines.**

**Figure S4. Transect cross-sections 8-9 for the southwestern Lake Mungo shoreline. Inset shows the location of these transects relative to the reconstructed main (ca. 70-71 m AHD) and mega-lake (ca. 75 m AHD) shorelines.**

Two additional transects were made in the southern transitional zone between the western, benched shoreline and the lunette, in the Joulni area (Figure S5). A well-defined lag of beach gravels extends for several hundred metres along this southern part of the lunette, near its crest. Ground truthing (transect 11) indicates that these gravels consistently occur at ca. 71 m AHD; combined with their stratigraphic position, these gravels most likely represent the main shoreline of the Mungo and Arumpo phases. The relatively low elevation of the lunette at this position of the lake shoreline, coupled with the shoreline reconstruction, suggests that it is possible that this region was inundated during the mega-lake phase, although further surveying and stratigraphic ground truthing is necessary to confirm this.

**Figure S5. Transect cross-sections for the southern Joulni area, transitional between the western Lake Mungo shoreline and its lunette. Transect 10 demonstrates the low elevation of the lunette at this position of the lake. Transect 11 shows the consistent elevation of beach gravels at ca. 70-71 m AHD.**

**Lake Shoreline reconstruction**

Shorelines were reconstructed on the basis of 3 arc-second SRTM DEMs and dGPS data obtained in the field, according to the process described below. All GIS work was performed in ArcGIS for Desktop®.

1. *Elevation data and accuracy:*

Three elevation datasets were available for study, namely:

1. 3 arc-second (~90m) SRTM (v4.1) digital elevation models (DEMs) provided by CIAT [1] and available for the entire region,
2. High-resolution (1m) DEMs derived from stereo aerial photographs and available for ~25.2 km^2^ on the eastern part of Lake Mungo, and
3. Differential GPS (dGPS) data collected in the field.

The latter two data sources were generated using the Geocentric Datum of Australia (GDA 1994) and the Map Grid of Australia (MGA) zone 54 projection, with elevations set relative to the Australian Height Datum (AHD). The SRTM DEMs were reprojected from WGS84 to GDA1994, MGA zone 54, using bilinear resampling with the ESRI Data Management Project Raster tool, so as to match the spatial reference of the other data sources. No misalignment resulting from faulty reprojection was detected.

The elevation values of the high-resolution DEMs closely match those obtained in the field over a distance of some 2.5km with a dGPS (69 data points along the WOC beach transect – see Figure S6); the DEM and dGPS elevations show a strong and positive linear correlation (r^2^ = .943, p < .001, n=69), and their maximum disagreement amounts to 66cm, with a mean and median difference of only 2cm (SD = 22cm) and 1.4cm (IQR = 29cm) respectively. The position and slope of the regression and equality (y=x) lines, shown in Figure S6, indicate some degree of bias in the datasets, whereby the elevation values derived from the DEMs tend to overestimate heights at lower elevations and underestimate heights at higher elevations relative to the dGPS data, but this bias is negligible.

**Figure S6. Accuracy of the available elevation data sources. A. SRTM DEM showing locations where the SRTM data overestimates or underestimates elevations relative to high-resolution (1m) AHD DEMs downsampled to match the spatial resolution of the SRTM DEM, as well as the location of the N-S, W-E, and WOC beach transects. B. Elevation profiles of the SRTM and downsampled AHD DEMs along the N-S transect, illustrating the systematic overestimation of elevations in the SRTM data, as well as the extent of the water levels on the SRTM DEM if: a) using the correct, corresponding AHD elevation value, b) applying a conservative 1m correction factor, and c) applying no correction factor. C. Elevation profiles of the SRTM and downsampled AHD DEMs along the W-E transect, illustrating the systematic overestimation of elevations in the SRTM data, as well as the extent of the water levels on the SRTM DEM if: a) using the correct, corresponding AHD elevation value, b) applying a conservative 1m correction factor, and c) applying no correction factor. D. Relationship between the elevation values of the SRTM and downsampled high-resolution (1m) AHD DEMs, illustrating both the systematic bias in the SRTM data and the overall strong correlation between the SRTM and the AHD DEM data. E. Relationship between the elevation values of the high-resolution (1m) AHD DEMs and the dGPS elevation values obtained in the field, showing the strong correlation between the two datasets and the lack of notable bias.**

The elevation values of the SRTM DEMs are problematic and show only a moderate correlation with the dGPS data points (r_s_ = .337, p = .005, n=69), as expected given the marked differences in spatial resolution and the relatively low vertical accuracy (~16m according to specifications) of the SRTM data. A preliminary assessment of the SRTM data and dGPS-derived elevations over transects located on the northern, western, and eastern parts of Lake Mungo strongly suggested that the SRTM data systematically overestimate elevations in this region. To test this and more reliably assess the accuracy of the SRTM data, the SRTM DEMs were compared with the high-resolution DEMs, shown above to be reliable proxies for dGPS elevations.

In order to compare the SRTM and high-resolution DEMs, the latter were downsampled to match the spatial resolution of the SRTM data using the ESRI Data Management Resample tool. Elevation data along two lines, drawn North-South and West-East and measuring ~3.1km and ~2.6km respectively (Figure S6a), were extracted from the SRTM and downsampled DEMs with the ESRI 3D Analyst Stack Profile tool and plotted in Gnuplot (http://www.gnuplot.info/). These elevation profiles were used to check that the data were not misaligned, and to illustrate the systematic overestimation of elevations in the SRTM data (Figure S6b,c). The elevation values from the downsampled DEM were then subtracted from the SRTM DEM using the ESRI Spatial Analyst Minus tool, creating a new difference raster.

The elevation values from the difference raster, which covers an area of ~25.2km^2^, indicate that the SRTM DEMs overestimate elevations over a cumulative area of ~23.8km^2^ (~94.4%) by an average of ~2.9m (SD=1.6) and underestimate elevations over a cumulative area of ~1.4km^2^ (~5.6%) by an average of ~1.1m (SD=1.1) (Figure S6a); overall, the mean and median differences between the SRTM and the downsampled DEMs is 2.7 (SD = 1.84) and 3 (IQR=2) meters respectively.

The elevation values from the downsampled DEMs and the SRTM DEMs exhibit a strong and positive linear correlation (r2 = .961, p < .001, n = 3350). The position and slope of the regression and equality (y=x) lines (Figure S6d) clearly indicates systematic bias in the SRTM data relative to the higher resolution DEM, consistently overestimating elevations, slightly more so at lower elevations than at higher elevations. The strong positive linear correlation between the two DEM sets and the relatively straightforward nature of the systematic bias in the SRTM data suggests that the SRTM data is suitable, with pertinent caveats, for the reconstruction of shorelines.

1. *Shoreline reconstruction:*

We created new water level rasters from the SRTM DEMs using the ESRI Spatial Analyst Map Algebra tool with conditional statements, and converted these to polygons using the ESRI Conversion Tools Raster to Polygon tool, with simplify polygons enabled. The resulting vector layers were cleaned by removing small, unconnected polygons. We opted to partially correct for the overestimation of the SRTM data by creating the water level rasters 1m higher than the dGPS derived heights of the shorelines, the equivalent of lowering the elevations of the entire SRTM raster dataset by the same amount and keeping the shoreline levels constant. The 1m correction factor is very conservative in light of the observed patterns (see Figure S6b,c), but it provides a reliable *minimum* estimate of the shorelines and thus better illustrates our main arguments than would a more aggressive correction factor.

The resulting water level vector layers provided reliable but visually inadequate results due to the low spatial resolution of the raster dataset. We therefore upsampled the SRTM data to a resolution of 5m with the ESRI Data Management Resample tool using bilinear resampling, and repeated the process described above, producing the final water levels shown in the various figures included in this paper. We compared the water level layers produced from the original SRTM data and the upsampled SRTM data and noticed no significant differences except in the quality of the visual display.

The same process was also repeated for the high-resolution AHD DEM in order to obtain actual water levels based on the dGPS elevations, but without correction factors.

*3. Lake volume calculation*

Lake areas for lake-full and mega-lake water levels were computed on the basis of the water level lake polygons using the ESRI Calculate Geometry tool. Since Lake Mungo and Lake Leaghur were connected during the mega-lake event, the portion of the mega-lake that corresponds roughly to Lake Mungo was isolated (Figure S7), and lake area was derived from the resulting polygon. For volume calculations the SRTM DEM was clipped to the extents of the two water level polygons, and the two resulting DEMs were converted to triangular irregular networks (TINs) using the ESRI 3D Analyst Raster to TIN tool. Volumes were then calculated on the basis of the TINs and the water level polygons using the ESRI 3D Analyst Polygon Volume tool.

**Table S2. Calculated area, volume and percentage change for the main and mega-lake phases.**

**Figure S7. Area used for estimating the area and volume of the Mungo mega-lake.**

**Sedimentology**

This section contains additional data relevant to the sedimentology component of this study, including a visual summary of the sedimentological characteristics of the different stratigraphic units, based on field photography and micromorphology collected from the central lunette transect (Figure S8); a summary of micromorphological analyses of the various stratigraphic units, based on the samples collected from the central lunette transect (Table S4); visual characteristics of the Red Lunette unit from both the central and northern parts of the lunette (Figure S9); particle size data for the Red Lunette facies transect, based on samples collected in the northern part of the lunette (Table S5); and particle size distribution for each of these samples (Figure S10).

**Table S3. Summary of the main characteristics and palaeoenvironmental interpretation for each late Quaternary stratigraphic unit observed within the lunette. The location, codes and ages for the OSL dating samples, including their position relative to the Red Lunette shoreline, are also given.**

**Figure S8. Sedimentological characteristics of the stratigraphic units present in the central and northern parts of the lunette, from youngest (top) to oldest (bottom).**

**Figure S9. Characteristics of the Red Lunette stratigraphic unit. A. In situ exposure of the Red Lunette gravel beach at 75 m AHD in the central part of the lunette, showing contacts between the underlying Upper Mungo and overlying Arumpo units. B. In situ exposure of the Red Lunette unit at 75 m AHD along the surveyed shorefront transect WOC 1. The beach gravels include non-carbonate rock gravels which indicate inflow of non-local clastic components. C. Lag surface at 73.5 m AHD in the northern part of the lunette, preserving non-local clastic components associated with the Red Lunette unit. D. Non-local clastic components collected from both in situ and lag surfaces associated with the 75 m AHD Red Lunette beach in both the northern and central parts of the lunette.**

**Table S4. Summary of sedimentary characteristics for the different stratigraphic units based on thin section micromorphology, in stratigraphic order from youngest to oldest.**

**Table S5. Particle size analysis data for the Red Lunette stratigraphic unit, northern lunette transect, relative to facies. Paired OSL dating is shown, where relevant; in the cases of PSA 1-3, however, the Red Lunette unit was too thin for OSL sample collection.**

**Figure S10. Bimodal particle size distributions, expressed as frequency percentage by volume, for the Red Lunette stratigraphic unit along the northern lunette transect.**

**Optically stimulated luminescence (OSL) dating**

This section contains additional data relevant to the OSL dating component of this study, including information pertaining to the single aliquot regenerative dose (SAR) measurement protocol; radial plots for the single grain measurements on each sample; overdispersion data (Table S6); and results from the finite mixture modeling on samples (Table S6).

Table S6 summarises the overdispersion values for each of the luminescence samples measured, giving an indication of the degree of scatter of equivalent dose from individual grains. Unsurprisingly, the overdispersion values for small aliquots were lower than those for single grains.

**Table S6. Overdispersion values for OSL samples. Single aliquot results are given in plain text, single grain results in italics. Red Lunette samples are highlighted in bold type.**

**Table S7. Results from finite mixture model analyses.**

Equivalent dose (D_e_) measurements were undertaken using an automated Risø TL-DA-20 reader with a single grain attachment. The D_e_ was measured on at least 600 individual grains of each sample using the single-aliquot regenerative-dose (SAR) protocol of [3, 4]. This comprised measurement of the natural signal followed by measurement of the OSL signal arising from four regenerative doses of increasing size, each followed by application of (and signal measurement from) a small test dose. In addition, a zero dose step and a final regenerative dose equivalent to the first applied dose were applied, the latter to assess the reproducibility (termed “recycling”) of dose response, and the former to assess potential transfer of charge (and therefore signal) by preheating during the measurement protocol (known as “thermal transfer”). Each protocol incorporated an infrared stimulated luminescence (IRSL) wash prior to each OSL signal measurement to identify potential feldspar contamination of the measured sample [4]. Preheat and cutheat temperatures of 260ºC and 220ºC respectively were determined based on the results of a preheat plateau test on sample EVA1112 (Figure S11).

**Figure S11. Preheat plateau test results for single aliquots of sample EVA1112. Dose response to preheat temperatures ranging between 180-280˚C was measured; the results indicate no dependence of dose on preheat temperature, and therefore the preheat and cutheat temperatures of 260ºC and 220ºC respectively were chosen for SAR measurements of dose on all samples.**

The following radial plots (Figures S12-S14) illustrate the equivalent dose distributions for the different samples.

**Figure S12. Radial plots of dose distributions for the central lunette beach samples, EVA1112-1115. Single aliquots are shown as open triangles and single grains as closed circles. The D_e_ for single aliquots is shown by the shaded grey band, and for single grains is shown as a solid black line. One exception is EVA1115, to which the finite mixture model was applied to the single grains. In this case, the different populations are shown by multiple black lines, with the thicker black line corresponding to the most likely age.**

**Figure S13. Radial plots of dose distributions for the central lunette backdune samples, EVA1116-1119. Single aliquots are shown as open triangles and single grains as closed circles. The D_e_ for single aliquots is shown by the shaded grey band, and for single grains is shown as a solid black line.**

**Figure S14. Radial plots of dose distributions for the northern lunette transect samples, EVA1255-1261. Since single grains only were measured for these samples, the radial plots show only single grain data as closed circles and the calculated D_e_ as a shaded grey band, with the exception of sample EVA1257, which was analysed using the finite mixture model. In the latter case, the different populations are shown by multiple black lines.**

**Figure S15. Radial plots of dose distributions for the channel sediment samples, EVA1265 and EVA1269. Since single grains only were measured for these samples, the radial plots show only single grain data as closed circles and the calculated D_e_ as a shaded grey band.**

To assess the suitability of the Lake Mungo quartz for luminescence dating using the SAR protocol, a dose recovery test, following [4], was undertaken on sample EVA1255 (Figure S16). Following laboratory bleaching, a laboratory beta dose of ~48 Gy was applied to 600 single grains (on 6 discs) of this sample, followed by application of the SAR protocol using the same parameters as used for the natural equivalent dose measurements. The resulting dose recovery ratio is 1.00 ± 0.01 with 12.9% overdispersion, calculated from 143 grains, indicating close to perfect dose recovery for all luminescent grains. This result supports the interpretation that the Lake Mungo sediments are highly suitable for luminescence dating using the SAR protocol.

**Figure S16. Radial plot summarising the results of the dose recovery test applied to sample EVA1255.**

**Table S8.** **Calculated concentrations of radioisotopes determined using high resolution germanium gamma spectrometry, analysed at VKTA Dresden. The gamma ray contribution to dose rates was determined from these data, using the conversion factors of [5]. Red Lunette data are shown in bold type.**

**Archaeology**

**Figure S17. Archaeological traces preserved within the RL, northern (island) transect. A. Baked sediment hearth complex, looking upslope and up-section. A ruler sits immediately to the left of the largest hearth. B. In situ lumps of baked sediment within the RL; modern aeolian drift obscures some of the surface. C. Baked sediment hearth traces, including the baked sediment depicted in image B. A lag of baked carbonate lumps and worked artefacts are also visible, and may derive from the RL. D. In situ burnt sediment hearth within the RL backdune sediments, exposed in cross section. E. Overview of RL exposures containing numerous hearths, looking towards the lake floor.**

**Figure S18.** **A. Exposure of two in situ baked sediment hearths within the RL, northern (island) transect. The hearths sit at two elevations with 13 cm of sediment accretion between them (elevations shown in the insets (B) and (C)), indicating repeated occupation of the island.**

**References**

1. Jarvis A, Reuter HI, Nelson A, Guevara E (2008) Hole-filled seamless SRTM data V4. International Centre for Tropical Agriculture (CIAT). Available: http://srtm.csi.cgiar.org.

2. Fitzsimmons KE, Stern N, Murray-Wallace CV (2014) Depositional history and archaeology of the central Lake Mungo lunette, Willandra Lakes, southeast Australia. Journal of Archaeological Science 41: 349-364.

3. Murray AS, Wintle AG (2000) Luminescence dating of quartz using an improved single-aliquot regenerative-dose protocol. Radiation Measurements 32: 57-73.

4. Murray AS, Wintle AG (2003) The single aliquot regenerative dose protocol: potential for improvements in reliability. Radiation Measurements 37: 377-381.

5. Adamiec G, Aitken M (1998) Dose-rate conversion factors: update. Ancient TL 16: 37-50.
