# Supplementary material for: The Mungo Mega-Lake Event, Semi-Arid Australia: Non-Linear Descent into the Last Ice Age, Implications for Human Behaviour
Source: PLoS One. 2015 Jun 17;10(6):e0127008. doi: 10.1371/journal.pone.0127008 (PMC4470511; doi:10.1371/journal.pone.0127008)
Supplement: S1 Table — (DOCX) [file pone.0127008.s020.docx]

**Table S1.** Summary of benching elevations representing shoreline erosion, indicating consistent elevations for the main and mega-lake shorelines.

| **Transect** | **Observed benching** | |
| --- | --- | --- |
|  | **Main shoreline (m AHD)** | **Mega-lake shoreline (m AHD)** |
| 1 | 71 | -^a, b^ |
| 2 | 72 | 75 |
| 3 | 70 | -^a, b^ |
| 4 | 69^c^ | 74 |
| 5 | 71^c^ | -^a^ |
| 6 | 70 | -^a^ |
| 7 | 69 | 74 |
| 8 | -^a^ | -^a^ |
| 9 | 69 | 74 |

^a^ No benching was observed at this location.

^b^ These transects were made at locations which would have been inundated during the mega-lake phase (see location inset; Figure S2). Therefore, the lack of elevation and benching to 75 m is consistent with the mega-lake shoreline reconstruction.

^c^ Benching at this location was poorly defined.
