# Supplementary material for: The Mungo Mega-Lake Event, Semi-Arid Australia: Non-Linear Descent into the Last Ice Age, Implications for Human Behaviour
Source: PLoS One. 2015 Jun 17;10(6):e0127008. doi: 10.1371/journal.pone.0127008 (PMC4470511; doi:10.1371/journal.pone.0127008)
Supplement: S2 Table — (DOCX) [file pone.0127008.s021.docx]

**Table S2.** Summary of the main characteristics and palaeoenvironmental interpretation for each late Quaternary stratigraphic unit observed within the lunette. The location, codes and ages for the OSL dating samples, including their position relative to the Red Lunette shoreline, are also given.

| **Stratigraphic Unit** | **Description** | **OSL samples** | **Site sampled** | **Position relative to Red lunette morphology** | **Age (ka)** |
| --- | --- | --- | --- | --- | --- |
| F/ Reactivated lunette | Aeolian sands on the crest and lee flanks of the lunette, overprinted by a characteristic brown sandy soil. | EVA1115 | Central lunette | Beach | 7.4 ± 0.8 |
| E/Arumpo | Alternating pale sands and clayey sands (containing pelletal clays), corresponding to oscillating lake levels. Contains multiple spatially discontinuous, weakly developed soils within various different beds throughout the sequence. | EVA1114,  EVA1118,  EVA1261 | Central lunette,  Central lunette,  Northern lunette | Beach,  Backdune,  Backdune | 23.6 ± 2.1,  22.0 ± 2.0,  22.9 ± 1.9 |
| D/ Red Lunette | Thin, steeply dipping red sandy unit, with beach pebbles on the lakeward flank, indicating permanent high lake levels. | EVA1113,  EVA1117,  EVA1257,  EVA1260 | Central lunette,  Central lunette,  Northern lunette, Northern lunette | Beach,  Backdune,  Foredune,  Backdune | 24.8 ± 2.2,  23.5 ±2.5,  22.5 ± 2.4,  24.1 ±3.5 |
| C/ Upper Mungo | Thin, discontinuously exposed alternating pale sands and clayey sands (containing pelletal clays), corresponding to oscillating lake levels. Weak, discontinuous brown soil. Contains hearths with fish remains. | EVA1112,  EVA1116,  EVA1255,  EVA1256,  EVA1258,  EVA1259 | Central lunette,  Central lunette,  Northern lunette, Northern lunette, Northern lunette, Northern lunette | Beach,  Backdune,  Beach,  Beach,  Backdune,  Backdune | 40.6 ± 4.2,  43.5 ± 4.0,  38.9 ± 3.2,  31.2 ± 3.3,  32.2 ± 4.2,  34.7 ± 5.1 |
| B/ Lower Mungo | Thin unit of red beach sands corresponding to permanent high lake levels. Weak, discontinuous brownish soil. Contains the oldest known human remains (Bowler et al. 2003). | EVA1119 | Central lunette | Backdune | 51.2 ± 9.9 |
