# Supplementary material for: The Mungo Mega-Lake Event, Semi-Arid Australia: Non-Linear Descent into the Last Ice Age, Implications for Human Behaviour
Source: PLoS One. 2015 Jun 17;10(6):e0127008. doi: 10.1371/journal.pone.0127008 (PMC4470511; doi:10.1371/journal.pone.0127008)
Supplement: S3 Table — The location, codes and ages for the OSL dating samples, including their position relative to the Red Lunette shoreline, are also given. (DOCX) [file pone.0127008.s022.docx]

**Table S3.** Summary of sedimentary characteristics for the different stratigraphic units based on thin section micromorphology, in stratigraphic order from youngest to oldest.

| **Sample** | **Stratigraphic unit** | **Equivalent to OSL sample (ka)** | **Shape^1^** | **Clast or matrix supported** | **Mineralogy^2^** | | | | | **Cutans^3^** |
| --- | --- | --- | --- | --- | --- | --- | --- | --- | --- | --- |
|  |  |  |  |  | **Q** | **Cl** | **FeOX** | **CaCO_3_** | **HM** |  |
| CMW7 | Arumpo | EVA1118 22.0 ± 2.0 | SR-R | Clast | X | CT, CP | - | - | - | P |
| CMW2 | Red lunette (beach) | EVA1117 23.5 ± 2.5 | SA-R | Clast | X | CT | X | - | - | P, R |
| CMW3 | Upper Mungo | EVA1116 43.5 ± 4.0 | SR-R | Clast | X | CP | - | - | - | C |
| CMW8 | Upper Mungo | EVA1116 43.5 ± 4.0 | R | Clast | X | CP | - | - | - | I |
| CMW1 | Upper Mungo | - | SA | Matrix (paleosol) | X | M | - | - | X | I |
| CMW4 | Lower Mungo | EVA1119 51.2 ± 9.9 | SR-R | Clast | X | CT | - | - | X | R, P |
| CMW6 | Lower Mungo | EVA1119 51.2 ± 9.9 | SA-SR | Clast | X | CT | X | X | X | R, P |
| CMW5 | Golgol | - | SA-SR | Matrix (paleosol) | X | M | X | X | - | I |

^1^ Abbreviations for shape descriptions: SA = subangular, SR = subrounded, R = rounded, A = angular.

^2^ Abbreviations for minerals: Q = quartz, F = feldspar, Cl = clay (as matrix, M; cutans, CT; clay pellets, CP), FeOX = iron oxide minerals, CaCO_3_ = calcium carbonate (pedogenic indicator), HM = heavy minerals (e.g. zircon, magnetite).

^3^ Abbreviations for cutan descriptions: I = intact cutans (pedogenic), P = partial cutans, R = relict cutans (non-pedogenic), C = clean grains, M = matrix development (Note: where more than one type of cutan is observed, the dominant type is listed first).
