# Supplementary material for: The Mungo Mega-Lake Event, Semi-Arid Australia: Non-Linear Descent into the Last Ice Age, Implications for Human Behaviour
Source: PLoS One. 2015 Jun 17;10(6):e0127008. doi: 10.1371/journal.pone.0127008 (PMC4470511; doi:10.1371/journal.pone.0127008)
Supplement: S4 Table — (DOCX) [file pone.0127008.s023.docx]

**Table S4.** Particle size analysis data for the Red Lunette stratigraphic unit, northern lunette transect, relative to facies. Paired OSL dating is shown, where relevant; in the cases of PSA 1-3, however, the Red Lunette unit was too thin for OSL sample collection.

| **Sample name** | | PSA 1 | PSA 2 | PSA 3 | PSA 4 | PSA 5 |
| --- | --- | --- | --- | --- | --- | --- |
| **Elevation (m AHD)** | | 72.7 | 73.0 | 73.6 | 75.5 | 78.8 |
| **Equivalent to OSL sample** | | - | - | - | EVA1257 22.5 ± 2.4 ka | EVA1260 24.1 ± 1.9 ka |
| **Position within Red Lunette** | | Shoreface, subaqueous | Shoreface, subaqueous | Shoreface, subaqueous | Beach | Backdune |
| **Proportion size classes** | **Sand (%)** | 50.7 | 60.8 | 77.9 | 76.6 | 84.0 |
|  | **Silt (%)** | 41.9 | 35.7 | 19.4 | 20.1 | 13.8 |
|  | **Clay (%)** | 7.4 | 3.6 | 2.6 | 3.4 | 2.2 |
| **Volume weighted mean (μm)** | | 139 (fine sand) | 170 (fine sand) | 239 (fine sand) | 161 (fine sand) | 263 (fine sand) |
| **Mode** | **1** | 252 | 261 | 264 | 177 | 291 |
|  | **2** | 18 | 28 | 22 | 13 | 16 |
|  | **3** | - | - | 1023 | - | - |
| **Mean ± 1σ (Ф)** | | 4.2 ± 2.4 | 3.4 ± 2.1 | 2.9 ± 1.9 | 3.3 ± 1.8 | 2.5 ± 1.7 |
| **Skew (Ф)** | | 0.21 | 0.41 | 0.47 | 0.44 | 0.59 |
| **Kurtosis (Ф)** | | 0.72 | 0.80 | 1.32 | 1.45 | 2.16 |
