# Supplementary material for: The Mungo Mega-Lake Event, Semi-Arid Australia: Non-Linear Descent into the Last Ice Age, Implications for Human Behaviour
Source: PLoS One. 2015 Jun 17;10(6):e0127008. doi: 10.1371/journal.pone.0127008 (PMC4470511; doi:10.1371/journal.pone.0127008)
Supplement: S5 Table — Paired OSL dating is shown, where relevant; in the cases of PSA 1–3, however, the Red Lunette unit was too thin for OSL sample collection. (DOCX) [file pone.0127008.s024.docx]

**Table S5.** Overdispersion values for OSL samples. Single aliquot results are given in plain text, single grain results in italics. Red Lunette samples are highlighted in bold type.

| **Sample code** | **De (Gy)** | **Overdispersion (%)** |
| --- | --- | --- |
| EVA1112 | 38.9 ± 1.1 | 13.0 |
| *EVA1112* | *36.8 ± 0.8* | *30.0* |
| **EVA1113** | **23.4 ± 0.6** | **11.0** |
| ***EVA1113*** | ***22.9 ± 0.4*** | ***26.3*** |
| EVA1114 | 24.4 ± 0.8 | 15.7 |
| *EVA1114* | *24.1 ± 0.8* | *33.2* |
| EVA1115 | 11.1 ± 1.0 | 42.8 |
| *EVA1115* | *All accepted grains* | *58.2* |
| *EVA1115* | *5.3 ± 0.2* | *20.0* |
| EVA1116 | 42.5 ± 1.6 | 18.2 |
| *EVA1116* | *39.8 ± 0.9* | *34.2* |
| **EVA1117** | **19.7 ± 0.5** | **12.7** |
| ***EVA1117*** | ***17.0 ± 0.3*** | ***23.0*** |
| EVA1118 | 21.2 ± 0.7 | 14.8 |
| *EVA1118* | *20.3 ± 0.4* | *14.8* |
| EVA1119 | 17.5 ± 0.7 | 10.0 |
| *EVA1119* | *17.4 ± 0.7* | *28.2* |
| *EVA1255* | *42.8 ± 1.1* | *27.0* |
| *EVA1256* | *23.4 ± 0.7* | *28.2* |
| ***EVA1257*** | *All accepted grains* | *25.8* |
| ***EVA1257*** | ***28.7 ± 2.0 (FMM)*** | ***11.0*** |
| *EVA1258* | *18.7 ± 0.5* | *24.0* |
| *EVA1259* | *17.3 ± 0.5* | *25.2* |
| ***EVA1260*** | ***21.6 ± 2.4*** | ***33.2*** |
| *EVA1261* | *23.4 ± 0.6* | *23.6* |
