# Supplementary material for: The Mungo Mega-Lake Event, Semi-Arid Australia: Non-Linear Descent into the Last Ice Age, Implications for Human Behaviour
Source: PLoS One. 2015 Jun 17;10(6):e0127008. doi: 10.1371/journal.pone.0127008 (PMC4470511; doi:10.1371/journal.pone.0127008)
Supplement: S6 Table — Single aliquot results are given in plain text, single grain results in italics. Red Lunette samples are highlighted in bold type. (DOCX) [file pone.0127008.s025.docx]

**Table S6.** Results from finite mixture model analyses.

| **Sample code** | **Number of components** | **De (Gy)** | **% population** | **BIC^1^** |
| --- | --- | --- | --- | --- |
| EVA1115 ^2^ | 3 | 3.2 ± 0.1 | 7 | 270 |
|  |  | 5.3 ± 0.2 | 47 |  |
|  |  | 12.9 ± 0.5 | 46 |  |
| EVA1257 ^3^ | 3 | 19.4 ± 1.2 | 28 | 32.0 |
|  |  | 28.7 ± 2.0 | 59 |  |
|  |  | 40.5 ± 2.0 | 13 |  |

^1^ This was the lowest value achieved with the model given a reasonable number of components and the small sample size. The small sample size reflects the low proportion of grains which passed the selection criteria, since most luminescent grains were saturated with respect to the dose-response curve.

^2^ Finite mixture modeling of this sample yielded a poor fit, even with 6 components. The 3-component result yielded the lowest BIC value, and suggests a very small proportion (7%) of very young grains, then an even split between the next youngest component (5.3 ± 0.2 Gy) and the oldest component (12.9 ± 0.5 Gy). The older component probably corresponds to sediments of Arumpo age (17.9 ± 2.0 ka) and is within error of the multiple grain result, as well as previously published ages for the Arumpo unit. The younger component most likely reflects the most recent reactivation phase, and therefore the true age of this unit (7.4 ± 0.8 ka). This latter reactivation age is consistent with that of the reactivated Arumpo unit near the crest of the lunette, as published in Fitzsimmons et al. 2014 (JAS), which is 8.3 ± 0.5 ka.

^3^ Finite mixture modeling of this sample yielded a good fit with 3 components. The majority of the grain population falls at 28.7 ± 2.0 Gy (59% of grains) and gives an age of 22.5 ± 2.4 ka, which is within error of the other ages calculated for the Red Lunette. The minor components indicate some mixing and residual dose.
