# Supplementary material for: The Mungo Mega-Lake Event, Semi-Arid Australia: Non-Linear Descent into the Last Ice Age, Implications for Human Behaviour
Source: PLoS One. 2015 Jun 17;10(6):e0127008. doi: 10.1371/journal.pone.0127008 (PMC4470511; doi:10.1371/journal.pone.0127008)
Supplement: S7 Table — (DOCX) [file pone.0127008.s026.docx]

**Table S7.** Calculated concentrations of radioisotopes determined using high resolution germanium gamma spectrometry, analysed at VKTA Dresden. The gamma ray contribution to dose rates was determined from these data, using the conversion factors of Adamiec and Aitken (1998). Red Lunette data are shown in bold type.

| **Sample code** | **K (%)** | **U (ppm)** | **Th (ppm)** |
| --- | --- | --- | --- |
| ***Red lunette beach – central lunette*** | | | |
| EVA1112 | 0.54 ± 0.08 | 0.54 ± 0.18 | 2.97 ± 0.14 |
| **EVA1113** | **0.51 ± 0.08** | **0.63 ± 0.09** | **3.18 ± 0.15** |
| EVA1114 | 0.62 ± 0.08 | 0.64 ± 0.10 | 3.18 ± 0.13 |
| EVA1115 | 0.35 ± 0.08 | 0.56 ± 0.10 | 2.46 ± 0.11 |
| ***Red lunette backdune – central lunette*** | | | |
| EVA1116 | 0.51 ± 0.08 | 0.66 ± 0.10 | 3.50 ± 0.16 |
| **EVA1117** | **0.35 ± 0.08** | **0.63 ± 0.10** | **2.54 ± 0.11** |
| EVA1118 | 0.52 ± 0.08 | 0.66 ± 0.09 | 3.27 ± 0.15 |
| EVA1119 | 0.12 ± 0.01 | 0.37 ± 0.08 | 1.04 ± 0.07 |
| ***Red lunette transect – northern lunette*** | | | |
| EVA1255 | 0.67 ± 0.08 | 0.74 ± 0.08 | 4.12 ± 0.09 |
| EVA1256 | 0.44 ± 0.08 | 0.52 ± 0.08 | 2.54 ± 0.09 |
| **EVA1257** | **0.60 ± 0.08** | **1.51 ± 0.08** | **5.79 ± 0.09** |
| EVA1258 | 0.25 ± 0.08 | 0.50 ± 0.08 | 2.12 ± 0.09 |
| EVA1259 | 0.25 ± 0.08 | 0.58 ± 0.08 | 1.66 ± 0.09 |
| **EVA1260** | **0.46 ± 0.08** | **0.77 ± 0.08** | **3.22 ± 0.09** |
| EVA1261 | 0.53 ± 0.08 | 1.00 ± 0.08 | 3.48 ± 0.09 |
